# Sequence Similarity Network Reveals Common Ancestry of Multidomain Proteins

**DOI:** 10.1371/journal.pcbi.1000063

**Published:** 2008-05-16

**Authors:** Nan Song, Jacob M. Joseph, George B. Davis, Dannie Durand

**Affiliations:** 1 Department of Biological Sciences, Carnegie Mellon University, Pittsburgh, Pennsylvania, United States of America; 2 Computational Biology, Carnegie Mellon University, Pittsburgh, Pennsylvania, United States of America; 3 School of Computer Science, Carnegie Mellon University, Pittsburgh, Pennsylvania, United States of America; University of Texas at Austin, United States of America

## Abstract

We address the problem of homology identification in complex multidomain families with varied domain architectures. The challenge is to distinguish sequence pairs that share common ancestry from pairs that share an inserted domain but are otherwise unrelated. This distinction is essential for accuracy in gene annotation, function prediction, and comparative genomics. There are two major obstacles to multidomain homology identification: lack of a formal definition and lack of curated benchmarks for evaluating the performance of new methods. We offer preliminary solutions to both problems: 1) an extension of the traditional model of homology to include domain insertions; and 2) a manually curated benchmark of well-studied families in mouse and human. We further present Neighborhood Correlation, a novel method that exploits the local structure of the sequence similarity network to identify homologs with great accuracy based on the observation that gene duplication and domain shuffling leave distinct patterns in the sequence similarity network. In a rigorous, empirical comparison using our curated data, Neighborhood Correlation outperforms sequence similarity, alignment length, and domain architecture comparison. Neighborhood Correlation is well suited for automated, genome-scale analyses. It is easy to compute, does not require explicit knowledge of domain architecture, and classifies both single and multidomain homologs with high accuracy. Homolog predictions obtained with our method, as well as our manually curated benchmark and a web-based visualization tool for exploratory analysis of the network neighborhood structure, are available at http://www.neighborhoodcorrelation.org. Our work represents a departure from the prevailing view that the concept of homology cannot be applied to genes that have undergone domain shuffling. In contrast to current approaches that either focus on the homology of individual domains or consider only families with identical domain architectures, we show that homology can be rationally defined for multidomain families with diverse architectures by considering the genomic context of the genes that encode them. Our study demonstrates the utility of mining network structure for evolutionary information, suggesting this is a fertile approach for investigating evolutionary processes in the post-genomic era.

## Author Summary

New genes evolve through the duplication and modification of existing genes. As a result, genes that share common ancestry tend to have similar structure and function. Computational methods that use common ancestry have been extraordinarily successful in inferring function. The practice of discerning evolutionary relationships is stymied, however, by modular sequences made up of two or more domains. When two genes share some domains but not others, it is difficult to distinguish a case of common ancestry from insertion of the same domain into both genes. We present a formal framework to define how multidomain genes are related, and propose a novel method for rapid, robust characterization of evolutionary relationships. In an empirical comparison with the current state of the art, we demonstrate superior performance of our method using a large hand-curated set of sequences known to share common ancestry. The success of our method derives from its unique ability to infer evolutionary history from local topology in the sequence similarity network. This represents a departure from the view that protein family classification must be restricted to families with conserved architecture. By exploiting the structure of the sequence similarity network, our approach surmounts this limitation and opens the door to studies of the role of modularity in protein evolution.

## Introduction

Accurate identification of *homologs*, sequences that share common ancestry, is essential for accuracy in function prediction and comparative genomics. Homology identification is integral to the annotation of novel genes [1] and prediction of gene function by various methods, including phylogenetic clustering [2], gene fusion analysis [3],[4], phylogenomic inference [5], and genomic context [6],[7]. Homologous genes are used as markers to identify homologous chromosomal regions for comparative mapping [8],[9], analysis of whole genome duplication [10],[11], phylogenetic footprinting [12], and operon prediction [13]–[15]. Pairwise homology detection is also an integral component of clustering approaches to protein family classification ([1],[16], and work cited therein).

All of these applications exploit one or both of the following properties of homologous sequences: genes that share common ancestry tend (1) to have similar structure and function, and (2) be located in homologous chromosomal regions, making them suitable markers for comparative genomics. Because of their prevalence and importance, it is desirable to incorporate multidomain sequences in such analyses: Multidomain proteins represent 40% of the metazoan proteome, with functional roles in signal transduction, cellular adhesion, tissue repair, and immune response [17]. However, multidomain sequences, especially those with promiscuous domains that occur in many contexts, are frequently excluded from genomic analyses due to lack of a theoretical framework and practical methods for detecting multidomain homologs. In this paper, we extend the traditional definition of homology [18] to multidomain sequences that share a common ancestral gene, providing a formalism suitable for modeling multidomain family evolution, design and validation of multidomain homology identification methods, and incorporation of multidomain sequences in genomic analyses.

**Figure 1:**
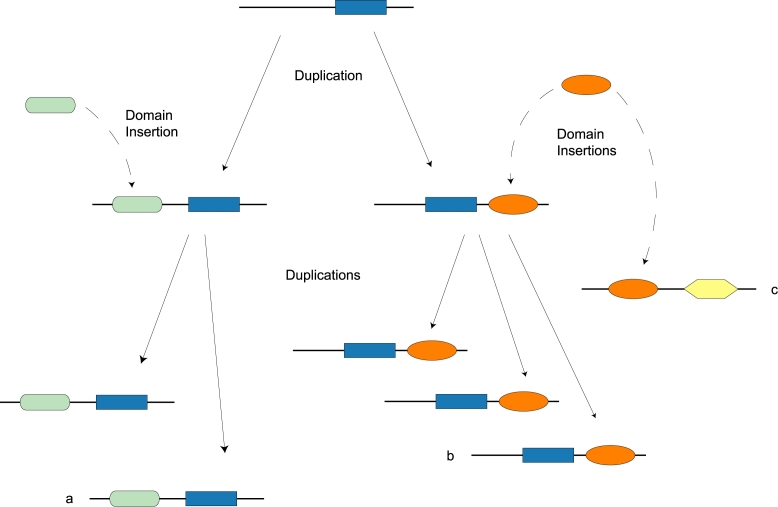
The evolution of a hypothetical multidomain family by gene duplication and domain insertion. Genes in the *a* and *b* subfamilies share a common ancestor but do not have identical domain composition. Gene *c* shares a homologous domain with genes in the *b* subfamily, but there is no gene that is ancestral to both *b* and *c*.

The original definition of molecular homology [18] does not capture multidomain evolution. Homology traditionally refers to evolution from a common ancestor by vertical descent (e.g., gene duplication and speciation), but multidomain proteins evolve via both vertical descent and domain insertion. For example, Figure 1 depicts two genes, *a* and *b*, which share not only a homologous domain but also a common ancestral gene. In contrast, *b* and *c* are a *domain-only match*, a pair of sequences that share similarity due to insertion of the same domain into both sequences but are otherwise unrelated.

**Figure 2:**
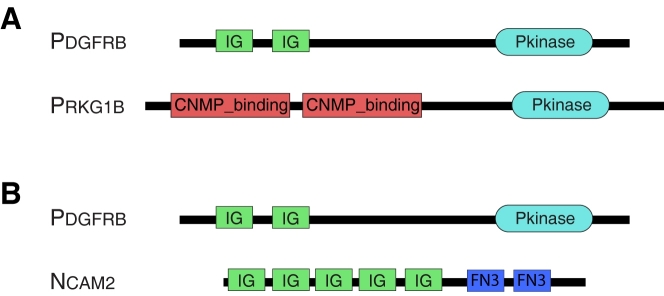
Domain models of a pair of multidomain homologs and a pair of sequences with a domain-only match. (A) Domain architectures of the multidomain homologs *PDGFRB* and *PRKG1B*. These sequences share a Pkinase domain, but have different auxiliary domains. (B) Domain architectures of *PDGFRB* and *NCAM2*, which have significant sequence similarity due to shared Ig domains, but do not share common ancestry.

Beta platelet-derived growth factor receptor (*PDGFRB*) and cGMP-dependent protein kinase 1, beta (*PRKG1B*), in Figure 2A, are enzymes involved in protein amino acid phosphorylation and provide a concrete example of this situation. Phylogenomic and structural evidence [19]–[22], as well as the promiscuity of the Ig and cNMP-binding domains, supports the common ancestry of this pair (see Methods). They have a statistically significant alignment with an E-value of 2.4*e* ^−8^ that covers 13% of the average of their lengths. While they share a common domain (Pkinase), the Ig domains are unique to *PDGFRB* and the cNMP-binding domains are unique to *PRKG1B*. An example of a domain-only match is shown in Figure 2B. Neural cell adhesion molecule 2 (*NCAM2*) and *PDGFRB* share two Ig domains, resulting in a significant alignment, also with an E-value of 2.4*e* ^−8^, and alignment coverage of 24%. However, the genes that encode them are not homologous and they perform different functions: *NCAM2* is involved in cell-cell adhesion with no enzymatic function.

The ability to distinguish multidomain homologs from unrelated pairs that share a domain is essential to genomic analysis. The evolutionary relationship between *a* and *b* in Figure 1 supports inferences about genome evolution, organization, and function. The same inferences would not necessarily be justified by the evolutionary relationship between *b* and *c*. For example, chromosomal regions enriched with homologous gene pairs are likely to be homologous themselves. In contrast, enrichment with homologous domains does not support the inference that a pair of chromosomal regions is homologous. Heuristics based on similarity and *alignment coverage* (the fraction of the mean sequence length covered by the optimal local alignment) have been proposed to screen out domain insertions. Recently, approaches based on domain architecture comparison have also been proposed [23]–[26]. To our knowledge, despite the prevalence of methods based on sequence similarity and alignment coverage [27]–[37], the accuracy of these heuristics has never been systematically tested. However, the examples in Figure 2 raise doubt about the general effectiveness of these methods. Both pairs have weak sequence similarity, short alignments, and a similar combination of shared and unique domains. Setting a significance threshold to eliminate *NCAM2* would also eliminate roughly 240 sequences that are related to *PDGFRB*, since more than a quarter of the Kinases that match *PDGFRB* have E-values less significant than 2.4*e* ^−8^. Alignment coverage would not help distinguish these two cases: the homologous pair has a *shorter* alignment than the unrelated pair. Nor could we separate this case by comparing domain content, since *PDGFRB* and *PRKG1B* share one domain, while *PDGFRB* and *NCAM2* share two. For this example, sequence similarity, the length of the shared region, and domain architecture comparison all fail to distinguish the homologous pair from the domain-only match.

To determine the extent of this problem, here we evaluate sequence similarity, alignment coverage, and domain architecture comparison on a hand-curated benchmark of 853,465 known homologous pairs. Our results show that these heuristics are all insufficient for consistent, reliable identification of multidomain homologs. Surprisingly, given its widespread use, even a modest alignment coverage requirement dramatically increased the number of mis-assigned homologs in our study. These results challenge two unstated, but widely accepted hypotheses: (1) homologous sequences share similarity along the bulk of their length and (2) the local alignment between homologous sequences usually covers a greater fraction of their mean length than the local alignments of sequences that only share a domain.

These observations suggest to us that sequences alone may not consistently contain enough information to differentiate homology from domain-only matches. We introduce a novel method, called *Neighborhood Correlation*, that leverages additional information contained in the weighted sequence similarity network to distinguish homologs from domain-only matches. In this network, each vertex corresponds to a sequence. Vertices whose corresponding sequences have significant similarity are connected by an edge with weight proportional to that similarity. The *neighborhood* of a sequence is the set of vertices adjacent to it; that is, the set of all sequences that match it above a predefined significance threshold. (In this work, “sequence neighborhood” refers to the local context of the sequence in the network and not to the region immediately surrounding it in the genome.) Our analysis demonstrates that the neighborhood structure of gene pairs related through shared domain insertions is characteristically different from that of pairs related through duplication or speciation. These differences in neighborhood organization are detectable and can be exploited to distinguish homology from domain sharing.

A homology detection method for genomic analysis must meet the following criteria: It should correctly predict homologous pairs and reject unrelated pairs, including those that share domains. With a single set of parameter values, it should perform reliably on sequences with a broad range of attributes, including single domain families, multidomain families, families with short regions of conservation, and families with weak sequence homology. Finally, it should be easy to use and fast enough for datasets comprising hundreds of millions of sequence pairs.

In an empirical evaluation, we demonstrate that Neighborhood Correlation meets these criteria. It is highly effective in classifying multidomain homologs and achieves superior performance in comparisons with sequence similarity (BLAST and PSI-BLAST), alignment coverage, and domain architecture comparison. To evaluate performance, we hand-curated a benchmark of 853,465 known homologous pairs of mouse and human sequences, drawn from twenty well-studied families. Our test set includes single-domain families, as well as multidomain families with promiscuous domains that are at risk for domain-only matches. Although comprehensive datasets are available for testing methods for predicting homology of *individual* domains [38],[39], we are unaware of any other gold-standard dataset of known multidomain families with variable domain architectures. We offer this validation dataset, which is based on published evidence by experts on each of the families, as a resource for future studies.

As a validation of our approach, we applied Neighborhood Correlation to all complete, mouse and human sequences in SwissProt 50.9 to predict homologs. A comparison of our predictions with the euKaryotic Clusters of Orthologous Groups (KOGs) database [40] showed that the set of protein sequences with highly correlated neighborhoods includes the vast majority of pairs that share an orthologous group (i.e., have the same KOG annotation). This is consistent with the fact that orthology is a more restrictive criterion than homology. We also show that most pairs in our set of predictions share at least one domain, according to the Pfam database [41], but many sequence pairs that share a domain are excluded. This is consistent with our goal of identifying *gene* homology rather than *domain* homology.

## Results

Homology has traditionally been defined in terms of families that evolve by vertical descent [18],[42]; that is, by speciation and gene duplication. However, multidomain sequences evolve by speciation, gene duplication, *and* acquistion of domains from outside the family [43] (Figure 1). The traditional definition of homology does not apply in this case, as previous authors have pointed out [42],[44]. In the words of Walter Fitch [42], “We must recognize that not all parts of a gene have the same history and thus, in such cases, that the gene is not the unit to which the terms orthology, paralogy, *et cetera*, apply.” It has been proposed that sub-genic sequence fragments should be the units of interest [44],[45]. However, there are many applications, such as ortholog detection, comparative mapping, and phylogenetic footprinting, for which it is essential to work with a definition of homology where the gene is the basic unit. Moreover, in order to study the evolution of multidomain gene families, it is necessary to focus on genes. The gene is the unit of selection. While domains confer modular function on genes, ultimately it is the functionality of those genes drives their retention.

### A Model of Multidomain Homology

**Figure 3:**
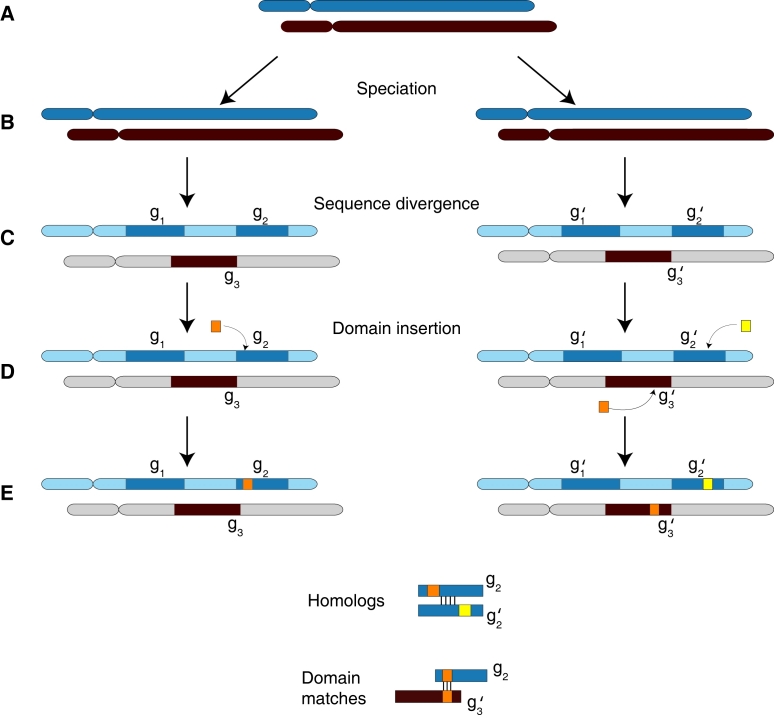
Evolutionary history of multidomain sequences in genomic context. (A) A hypothetical genome with two chromosomes. (B) Both chromosomes are copied through duplication or speciation, resulting in two identical copies. (C) Following sequence divergence, similarity is only retained in coding regions. (D) Two instances of the orange domain are inserted in *g*_2_ and *g*_3_’, respectively. A yellow domain is inserted in *g*_2_’. (E) Conserved genomic context shows that genes *g*_2_ are *g*_2_’ are homologous genes, although they contain unrelated domains. Similarly, genes *g*_2_ and *g*_3_’ contain homologous domains, but are not homologous genes.

Here, we propose a model of multidomain homology based on vertical descent and insertion of a sequence fragment into an existing gene. In our model, two sequences are homologous if they are encoded by genes that share an ancestral *locus*. The rationale for this definition is illustrated in Figure 3, which shows the evolution of genes through vertical descent and domain insertion in the context of the chromosomes in which they reside. When genomic context is taken into account, it is clear that genes *g*_2_ and *g*_2_′ are homologous, despite the fact that *g*_2_ contains a domain not present in *g*_2_′ and vice-versa. In contrast, genes *g*_2_ and *g*_3_′ are *not* homologous, despite the fact that they share a homologous domain, since *g_2_* and *g*_3_′ are not located in chromosomal regions that share common ancestry. For comparative mapping applications, where homologous genes are used as markers for identifying chromosomal regions, this distinction is crucial. For example, phylogenetic footprinting [12] predicts transcription factor binding sites by identifying homologous genes and then searching their flanking chromosomal regions for conserved sequence motifs. In Figure 3, the regions upstream of *g*_2_ and *g*_2_′ have an elevated probability of sharing conserved motifs since they share common ancestry. However, there is no reason to expect an enrichment of motifs shared between the flanking regions of *g_2_* and *g*_3_′.

Our model is applicable to families that evolved through acquisition of a new domain by an existing gene. This can occur through insertion of sequence fragments into the gene or by recruitment of adjacent exons. Formation of a new gene architecture by domain loss is also consistent with our model. Several lines of evidence suggest that acquisition of an auxiliary domain by an existing gene is a relatively common mode of domain shuffling. First, a substantial number of metazoan, chordate, and vertebrate families have been identified that evolved through a pattern of duplication, insertion of domains, and further duplication, a pattern consistent with this model [46],[47]. Second, the existence of promiscuous domains that lend themselves to insertion in new chromosomal environments [48],[49] supports an insertion model. Third, domain insertion is more likely to be successful when a domain is inserted into an existing functional environment, e.g., into the intron of an existing gene. In this case, all regulatory and termination signals required for successful transcription are already present. A fourth line of evidence stems from analyses of the flanking DNA of genes that arose very recently, where traces of the particular domain shuffling mechanism that occurred can still be observed. A number of recently evolved metazoan genes have been discovered that arose through duplication of an existing gene, followed by acquisition of one or more domains by unequal crossing over or by retrotransposition [50]–[54]. Finally, a number of studies have inferred relative rates of various domain shuffling events by applying parsimony models to abstract domain architectures. Their results suggest that the most common domain shuffling scenario involves insertion or deletion of a single domain into an existing multidomain architecture [24],[55],[56].

Our model is not applicable to the case where a new domain architecture is assembled *de novo* from several unrelated building blocks and subsequently acquires a regulatory region. We consider such a novel architecture to be the progenitor of a new family, since it is not clear that the ancestry of any one constituent is preferred. Similarly, our model does not capture formation of new architectures through fragmentation of more complex ones. However, recent evidence suggests that both of these scenarios occur rarely [24],[55],[57].

### Neighborhood Correlation

Homology detection is the problem of distinguishing between sequence pairs with different types of evolutionary histories: evolution via gene duplication or via domain insertion. Sequence similarity, alignment coverage, and domain architecture comparison have all been considered for this purpose. However, none of these distinguish the homologous pair from the domain-only match given in Figure 2. The empirical results in the following sections confirm that this is not an isolated example. Accurate classification of multidomain homologs requires additional information from another source.

The structure of the sequence similarity network provides a basis for distinguishing pairs related through vertical descent from other pairs. The local network neighborhoods of homologs and domain-only matches differ in both topology and edge weights. In particular, for homologous pairs, the shared neighborhood (i.e., the set of vertices adjacent to both members of the pair) tends to have more vertices and stronger edge weights than their unique neighborhoods (i.e., vertices adjacent to one pair but not the other). This is not true for domain-only matches. We express this distinction quantitatively by the Neighborhood Correlation score of two sequences, defined to be the correlation coefficient of their respective neighborhoods:
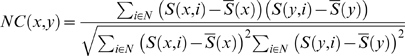
(1)where *S(x,i)* is the normalized bit score [58] of the optimal local alignment of query sequence *x* and database sequence *i*, *N* is the number of sequences in the database, and 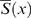 is the mean of *S(x,i)* over all sequences (see Methods). Note that *NC*(*x*,*y*) increases with the number, weight, and correlation of edges in the shared neighborhoods of *x* and *y* and decreases with the number and weight of edges in their unique neighborhoods.

The Neighborhood Correlation score captures properties of the sequence similarity network that are strongly influenced by the evolutionary processes of interest. The number of edges in the shared and unique neighborhoods is influenced by the rates of gene duplication and domain insertion, while edge weights depend on sequence divergence. Immediately following a gene duplication, the two resulting paralogs have identical neighborhoods. The Neighborhood Correlation score of this new pair is initially one and decreases as the sequences diverge. Additional gene duplications in the same family further increase the size of the shared neighborhood and, hence, the Neighborhood Correlation score. In contrast, if a domain is inserted into a single member of the pair, the number of edges in its unique neighborhood increases and the Neighborhood Correlation score decreases. The increase in the number of unique edges is directly related to the promiscuity of the inserted domain, while the weights of these new edges are proportional to the degree of sequence conservation in the domain superfamily. In practice, the impact of insertion of a domain into a single member on the Neighborhood Correlation score is typically small because promiscuity and sequence conservation within domain superfamilies are inversely related. For example, Pfam domains exhibit a highly significant, negative correlation between domain promiscuity (see Methods) and sequence identity (ρ = −0.21, *p* = 2.08*e* ^−30^, Spearman test). This can be understood by observing that when a domain is inserted into a new context, it is likely to experience new selective pressures leading to rapid mutational change.

**Figure 4:**
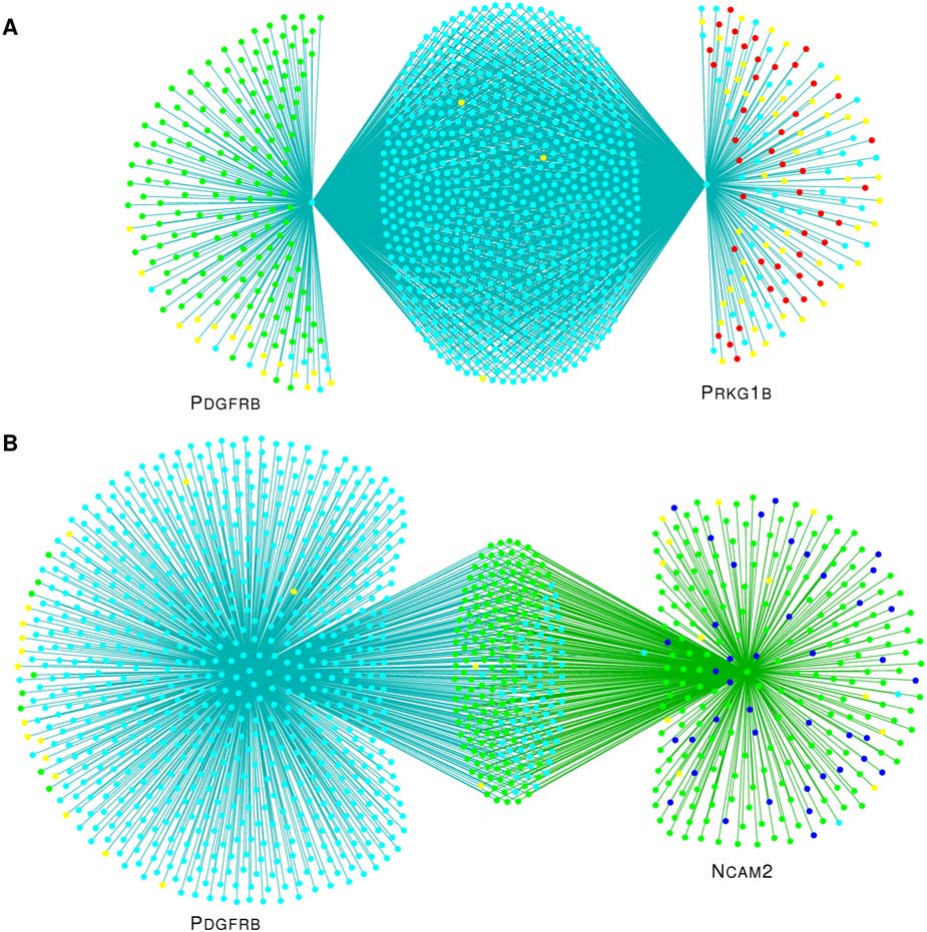
Differences in neighborhood structure of the sequence similarity network reflect differences in evolutionary history. Network neighborhoods in which nodes represent sequences. Edges connect pairs with significant sequence similarity. Edge weights reflecting degree of sequence similarity are not shown. (A) The neighborhoods of the homologous pair, *PDGFRB* and *PRKG1B*. *PDGFRB* and *PRKG1B* share 779 neighbors, mostly Kinases (turquoise nodes). These are strong matches due to a shared kinase domain. *PDGFRB* has 183 unique neighbors, mostly due to weak matches with Ig domains (green nodes). *PRKG1B* has 142 unique neighbors due to weak matches with the cNMP-binding domain (red nodes). Other matching sequences are shown in yellow. (B) *PDGFRB* and *NCAM2*, a domain-only match, have 232 matches in common. *PDGFRB* has 730 unique neighbors and *NCAM2* has 240, mostly due to Fn3 domains (dark blue nodes).

To see how these principles play out in practice, we consider the neighborhoods of *PDGFRB*, *PRKG1B*, and *NCAM2* in the sequence similarity network derived from our test dataset (Figures 2 and 4). Although the homologous pair, *PDGFRB* and *PRKG1B*, and the domain sharers, *PDGFRB* and *NCAM2*, have pairwise alignments with similar properties (E-value, alignment length, number of shared domains), their neighborhoods in the weighted sequence similarity network are very different. The shared neighborhood of the Kinase homologs *PDGFRB* and *PRKG1B* is substantially larger (779 sequences) than their unique neighborhoods (183 and 142 sequences, respectively). The shared neighborhood consists almost entirely of Kinases. The unique neighborhoods are dominated by domain-only matches, due to Ig in the case of *PDGFRB* and the cNMP-binding domain in the case of *PRKG1B*. Sequence similarities within these unique neighborhoods are weak; the Pfam models for the Ig and cNMP-binding domains have average sequence identities of 20% and 18%, respectively. Thus, the edge weights (not shown) in the shared neighborhood are strong and well correlated, while the edge weights in the unique neighborhoods are weak, yielding a Neighborhood Correlation score of *NC* = 0.65.

Conversely, *PDGFRB* and *NCAM2* are related through domain insertion and have significant sequence similarity due to a shared Ig domain. Their shared neighborhood is relatively small (242 sequences) and comprised primarily of Ig-based matches. These contribute little to the Neighborhood Correlation score of this pair due to low sequence conservation within the Ig superfamily. In contrast, the unique neighborhood of *PDGFRB* is large (630 sequences), with strong edge weights. For these reasons, *PDGFRB* and *NCAM2* have a Neighborhood Correlation score of 0.29, distinctly smaller than the score for *PDGFRB* and *PRKG1B*. Unlike sequence comparison, this clear difference in neighborhood structure can be used to recognize multidomain homology.

### A Benchmark Dataset for Multidomain Homology

Evaluation of classification performance requires a trusted set of positive examples (known homologous pairs) and negative examples (pairs known not to share common ancestry). Although benchmarks are available for detection of remote homology (e.g., SCOP [38], CATH [39]), functional similarity (e.g., the Gene Ontology (GO) [59]), orthology (e.g, COGs [40]), and structural genomics ([16],^45^,[60], and work cited therein), we are unaware of any gold-standard validation dataset for multidomain homology. Our benchmark is designed to be suitable for testing two classification goals: good overall performance on a large set of sequence pairs and consistent performance on individual families with varying properties. To satisfy these needs, we constructed a test set of 1577 sequences from 20 families of known evolutionary origin (Table 1). The families encompass a broad range of functional categories, summarized in Table 2. The full curation procedure is described in Methods and Text S1.

**Table 1:** Test family statistics.

Family	*k*
*ALL*	1577
*ALL-Kin*	671
Single domain families	
ACSL	10
FGF	44
FOX	81
Tbox	31
TNF	32
USP	77
WNT	38
Multidomain families: conserved architecture	
DVL	7
GATA	12
Notch	8
KIR	14
TRAF	12
Multidomain families: variable architecture	
ADAM	44
Kinase	906
Kinesin	56
Laminin	22
Myosin	46
PDE	44
SEMA	38
TNFR	55

*k*: the number of sequences.

**Table 2:** Functional properties of the 20 test families.

Functional category		Family
Biological process	Neural development	SEMA, Notch
	Immune response	TNF, TNFR, KIR
	Development and homeostatic regulation	ADAM, FGF, WNT
	Cell-cell/cell-matrix interaction	ADAM, Laminin, Notch
Molecular function	Transcription factor	FOX, GATA, Tbx
	Intracellular signal transducer	Kinase, DVL, TRAF
	Enzyme	ACSL, ADAM, Kinase, USP, PDE
	Motor	Myosin, Kinesin
	Structural molecule	Laminin
	Ligand	FGF, SEMA, TNF, WNT
	Receptor	TNFR, KIR, Notch
Cellular location	Extracellular	ADAM, FGF, Laminin, SEMA, WNT
	Transmembrane	ADAM, SEMA, KIR, Kinase, Notch, TNF, TNFR
	Intracellular	ACSL, DVL, FOX, GATA, Myosin, Kinesin, PDE, Tbx, Kinase, TRAF,USP

For each family, we identified two sets of sequence pairs: family (FF) pairs, where both members of the pair are in the family, and non-family (FO) pairs, where only one of the two sequences is in the family. Given a family of size *k*, we obtain *k* ^2^ FF pairs (the positive examples) and *k*(*N*−*k*) FO pairs (the negative examples). Individual families, which cover a range of functional properties and domain architecture complexity, can be used for family specific tests. In addition, we constructed a test set (*ALL*) for general performance evaluation by merging all sets of FF and, respectively, FO pairs, yielding 853,465 positive and 40,459,204 negative examples. Performance measurements obtained with this set could be biased by the Kinase family, which is much larger than the other families. We therefore also considered the set of all sequences excluding the Kinases (*ALL-Kin*), resulting in 32,629 positive and 17,545,558 negative examples.

Our goal is a method that can correctly identify homologs in multidomain families without degrading performance in other types of families. We therefore devised a benchmark to test a range of homology detection challenges, involving single domain as well as multidomain families. Families with complex and varied domain architectures represent the primary challenge undertaken in this study. Such families result from duplication, domain accretion, and further duplication. Some of these families are defined by a single domain that is unique to the family (e.g., Kinase), while others are characterized by a particular combination of domains (e.g., ADAM) or by a conserved set of domains with variations in domain copy number (e.g. Laminin). Modularity in both single and multidomain families can also arise through the presence of sequence motifs, such as subcellular localization signals, transactivation sequences (e.g., Tbox), and functional components that confer substrate specificity (e.g. USP). These motifs can result in matches to unrelated sequences. In addition, promiscuous domains challenge homology identification because they can result in significant sequence similarity but carry little information about gene homology. Promiscuity can confound reliable detection of homologs even in families with conserved domain architectures.

Remote homology detection is a serious challenge that has received widespread attention. In our dataset, this challenge is represented set by FGF, TNF, TNFR, and USP, families that exhibit low sequence conservation. Finally, we considered homologous pairs with short conserved regions. A minimum alignment coverage criterion is frequently imposed to eliminate domain-only matches, reflecting a widely held, but untested belief that homologous pairs have regions of similarity that cover a substantial fraction of their length. To test the robustness of homology detection methods with respect to alignment length, we included single domain families with short conserved regions such as the Tbox family.

Our selection of test families was limited to those for which it was possible to obtain evidence concerning their evolutionary history. Evolutionary evidence was obtained from published articles and/or curation by a nomenclature committee. In the best cases, direct syntenic evidence of vertical descent can be found. In other cases, indirect evidence such as conserved intron/exon structure is used. Phylogenetic evidence can confirm vertical descent, for example, if all domains in a family have consistent phylogenies. However, phylogenetic disagreement between core and auxiliary domains does not rule out homology according to our model. For each, the evidence used is described in Text S1.

### Accuracy of Homolog Identification

We evaluated Neighborhood Correlation using our benchmark, and compared its performance with other methods currently in use. We considered performance on multidomain homology identification, as well as overall performance on diverse, heterogeneous datasets. We also used Neighborhood Correlation to predict novel homologous relationships.

### Methods Compared

We compared the performance of Neighborhood Correlation with BLAST [61], alignment coverage [27], and PSI-BLAST [58], methods commonly used for assessing homology, as well as Domain Architecture Comparison (DAC), a recently introduced approach that compares sequences by considering their constituent domains [23]–[26],[55].

BLAST gives a measure of sequence similarity based on the optimal local alignment between two sequences. BLAST does not capture gene structure (e.g., domain organization), nor does it reflect additional information that might be derived from suboptimal local alignments. BLAST is widely used, its behavior is well understood, and its scores are easily compared with those from other studies. A great deal of attention has been devoted to tuning BLAST performance and to developing accurate statistical tests. It represents an attractive balance between rigor and speed.

A significant BLAST score is evidence of similarity greater than that expected by chance, but cannot distinguish whether that similarity stems from vertical descent or domain insertion. In order to eliminate domain-only matches, many analyses combine sequence similarity with alignment coverage to identify homologs [28]–[37]. To be considered homologous, sequence pairs must then satisfy a second criterion in addition to significant sequence similarity: the fraction of the sequence length covered by the optimal local alignment must meet a pre-specified threshold. To our knowledge, alignment coverage criteria have never been empirically evaluated. In this work, we demonstrate that such a requirement is highly detrimental to performance overall, and in nearly all tested families.

In the presence of high sequence divergence, BLAST is limited by the amount of information that can be derived from pairwise comparison. To address this problem, approaches based on multiple sequence alignments (MSAs) have been used to increase sensitivity. PSI-BLAST, one of the most widely used examples of this approach, constructs a Position Specific Scoring Matrix (PSSM) through iterative search and has been shown to dramatically improve sensitivity [62]. MSA-based methods are designed to detect remote homology, not multidomain homology. Since sequences with different architectures cannot be aligned, MSA-based methods are not a natural choice for multidomain homology detection. We included PSI-BLAST in our study because it is widely used as a standard for remote homology detection.

In addition to sequence based methods, we considered direct comparison of domain architectures for multidomain homology detection. Each sequence was represented by a linear sequence of Pfam domains. Linker sequences between domains were ignored, as was sequence variation between instances of a given Pfam domain family. The resulting domain architectures were compared based on their domain composition. In a previous study, we proposed and evaluated 21 different methods for comparing domain architectures [23]. These methods considered properties such as the number of shared domains, domain copy number, total number of domains in a protein, domain order, and domain promiscuity. We included the domain architecture comparison strategy that exhibited the best performance from that study in our current study. This method assigns a score to each pair based on the number of shared domains (see Methods), following the rationale that homologous pairs will have more domains in common than pairs related through domain insertion. In assessing similarity, each domain is assigned a weight inversely proportional to its promiscuity. This reflects the assumption that rare domains convey more information about homology than promiscuous domains.

### Evaluation Procedure

The performance of each method was assessed via the *ROC-n* score (Table 3), which represents both false positives and false negatives (see Methods). *ROC-n* is the area under the Receiver Operating Characteristic (*ROC*) curve comprised of the top ranking pairs up to the first *n* false positives. We used *n* = 100*k*, where *k* is family size, corresponding to 100 false positives per query.

**Table 3:** *ROC-100k* scores for Neighborhood Correlation, BLAST, PSI-BLAST, and Domain Architecture Comparison for all families.

	NC		BLAST	p-value	PSI-BLAST	p-value	DAC	p-value
*ALL*	**0.8148**		0.5838	0	0.7080	0	0.4431	0
*ALL-Kin*	0.8353		0.7505	0	0.7375	0	**0.8960**	0
Single domain families								
ACSL	**1.0000**	**1.0000**		-	**1.0000**	-	0.8184	0
FGF	**1.0000**	0.9920		0	**1.0000**	-	**1.0000**	–
FOX	**1.0000**	0.9996		0	0.9985	1.3e-04	0.9756	0
Tbox	**1.0000**	**1.0000**		-	**1.0000**	-	0.9376	0
TNF	0.3992	0.3631		0	0.6764	0	**1.0000**	0
USP	0.9236	0.8666		0	**0.9856**	0	0.9395	0
WNT	**1.0000**	**1.0000**		-	**1.0000**	-	**1.0000**	–
Mean	0.9033	0.8888			0.9515		**0.9530**	
Multidomain families: conserved architecture								
DVL	**1.0000**		**1.0000**	-	**1.0000**	-	**1.0000**	–
GATA	**1.0000**		**1.0000**	-	**1.0000**	-	0.9675	–
Notch	**1.0000**		**1.0000**	-	**1.0000**	-	**1.0000**	–
KIR	**1.0000**		0.9971	2.0e-15	0.9876	4.4e-16	**1.0000**	–
TRAF	**1.0000**		**1.0000**	-	**1.0000**	-	0.9843	2.2e-16
Mean	**1.0000**		0.9994		0.9975		0.9904	
Multidomain families: variable architecture								
ADAM	**1.0000**		0.9830	0	0.9061	0	0.9552	0
Kinase	**0.8362**		0.6164	0	0.7238	0	0.3789	0
Kinesin	0.9757		0.9806	-	**0.9866**	8.5e-12	0.9640	0
Laminin	**0.9592**		0.9245	0	0.8028	0	0.9055	0
Myosin	0.8046		**0.9870**	0	0.9796	0	0.8435	4.4e-16
PDE	**0.7565**		**0.7565**	-	0.7562	0	0.7174	0
SEMA	**1.0000**		0.9983	1.1e-06	0.9986	1.3e-04	**1.0000**	–
TNFR	**0.6909**		0.5607	0	0.6278	0	0.5390	0
Mean	**0.8779**		0.8509		0.8477		0.7879	

The maximum value in each row is shown in bold. The significance of the difference of the *ROC-100k* score for each method compared with that of Neighborhood Correlation is expressed as a p-value. Dashes indicate *ROC-100k* scores that are not significantly different at the 0.001 level.

In evaluating homology identification methods, we consider two user models. Genome-scale analyses require all-against-all comparison of a large and heterogeneous set of sequences. In order to be suitable for automated, genomic analyses, a method must be robust enough for use without human intervention, deliver consistent behavior on different types of domain architectures, and be fast and easy to use. In this case, the goal is to maximize the total number of homolog pairs that are correctly predicted. A second application is analysis of individual families, where the goal is to obtain good per-family prediction scores over a wide range of families.

To evaluate performance for both user models, we report *ROC-100k* scores for all pairs (*ALL* and *ALL-Kin*), as well as *ROC-100k* scores for each family. To show how the methods tested behave on proteins with various attributes, we also report the average *ROC-100k* score per family for single domain families, multidomain families with conserved architectures, and multidomain families with variable architectures.

**Figure 5:**
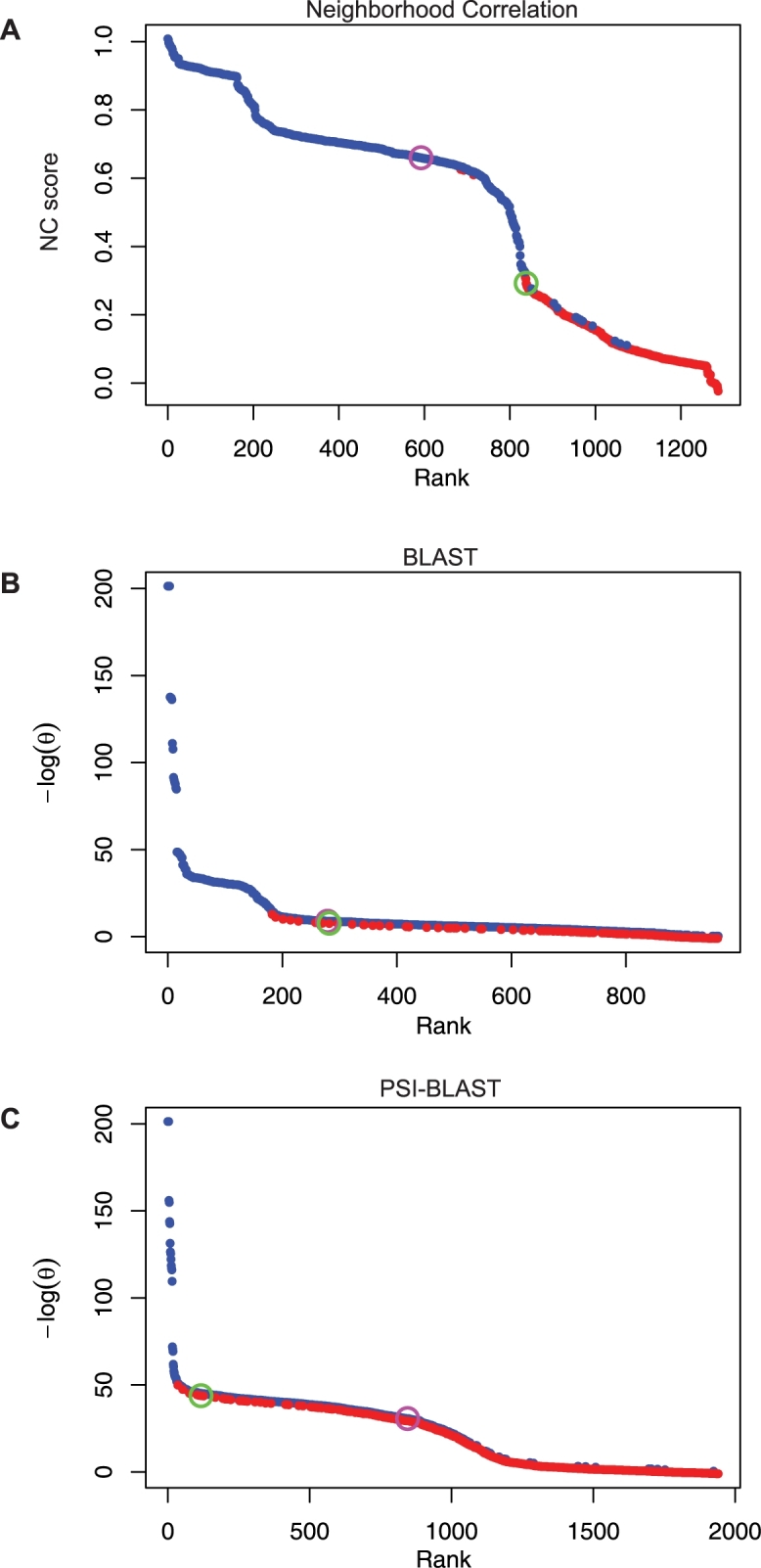
Rank plots for the query sequence *PDGFRB*. Family and non-family matches are shown in blue and red, respectively. Matches with the Kinase *PRKG1B* and the non-Kinase *NCAM2* are indicated by magenta and green circles. Scores of matching sequences ranked by (A) Neighborhood Correlation score, (B) BLAST score, and (C) PSI-BLAST score.

As a visualization tool, we generated *rank plots*, which show the scores of all matches to a given query sequence in rank order. Rank plots provide a visual representation of the organizational structure of the network neighborhood of the query sequence, as well as organizational substructure within the family. For example, Figure 5 shows a rank plot for the query sequence *PDGFRB*, a protein tyrosine kinase. The break in the curve in Figure 5B at *NC*≈0.8 corresponds to the first match to a Serine/Threonine Kinase, the inflection point at *NC*≈0.75 corresponds to the first match to a Dual-Specificity Kinase, and the downward plunge at *NC*≈0.59 corresponds to the first Casein Kinase. Rank plots for each of the 26,197 sequences in our dataset are provided at http://www.neighborhoodcorrelation.org.

### Neighborhood Correlation Performance

When all considered classifiers are applied to the aggregate set of sequence pairs (*ALL*), Neighborhood Correlation dramatically outperforms the other three methods (Table 3, Figures S1 and S2). In the *ALL-Kin* dataset, Neighborhood Correlation yields better performance than BLAST and PSI-BLAST, but performs slightly worse than DAC. The superior performance of Neighborhood Correlation on the *ALL* and *ALL-Kin* datasets demonstrates that its optimal classification threshold is less sensitive to family specific properties than those of BLAST or PSI-BLAST.

When performance on individual families is considered, Neighborhood Correlation is generally more robust than the other three methods. It perfectly classifies twelve families, more than any other method. In addition, in 16 of 20 families, the discriminatory performance of Neighborhood Correlation is better than or equal to that of all other methods. In particular, Neighborhood Correlation obtains the highest average score for both conserved and variable architectures and performs much better on individual multidomain families except for Myosin and Kinesin. For families with high sequence divergence, including FGF, TNF, and USP, Neighborhood Correlation performs better than BLAST, indicating that neighborhood structure can compensate for a low signal to noise ratio in pairwise comparisons of remote homologs. PSI-BLAST also performs well in such cases.

**Figure 6:**
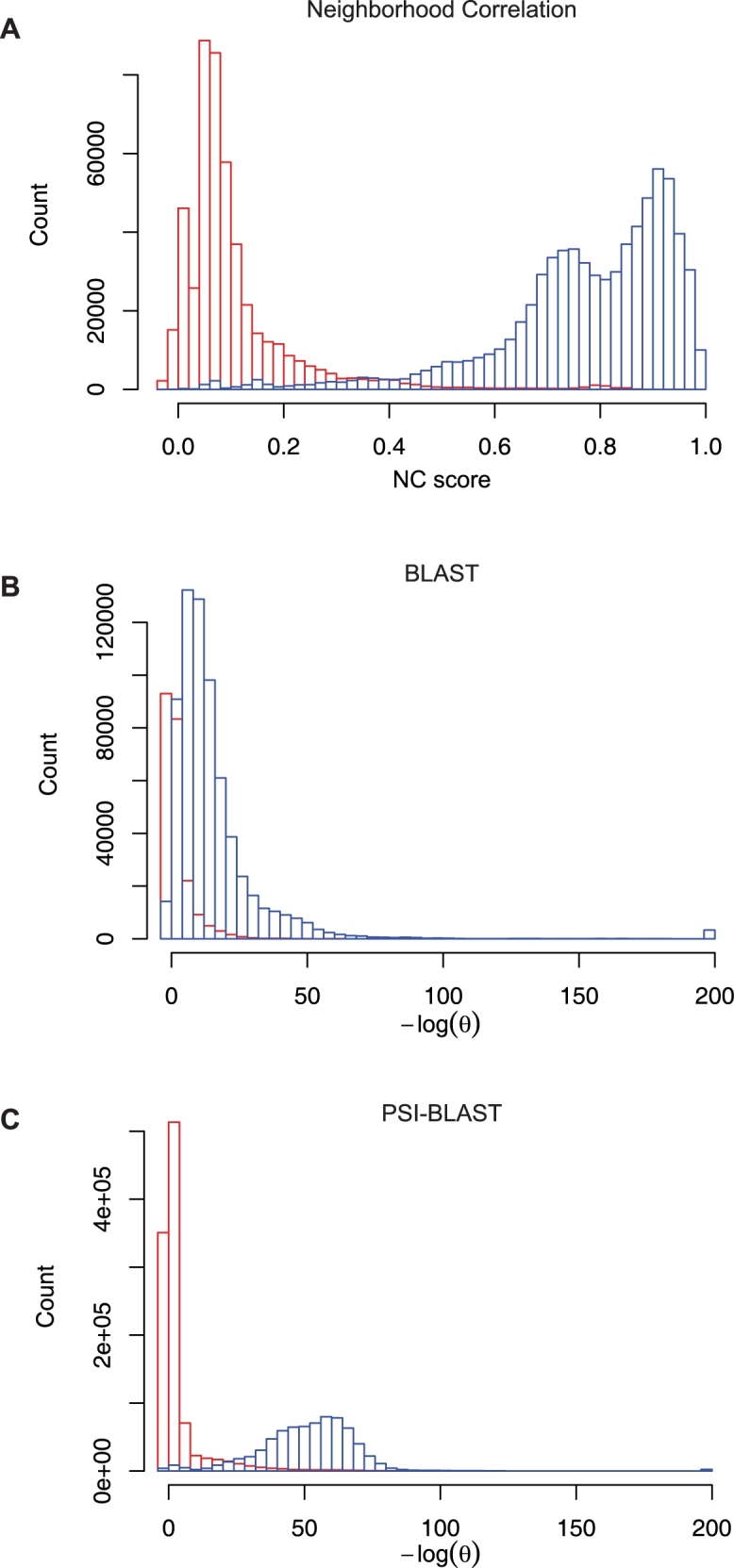
Distribution of scores for all family and non-family pairs in the Kinase family. Family and non-family matches are shown in blue and red, respectively. (A) Neighborhood Correlation scores, (B) BLAST scores, and (C) PSI-BLAST scores.

To demonstrate why Neighborhood Correlation is more effective for complex families, we consider its performance on the Kinase family. Figure 5 shows a rank plot of the results of a query with the Kinase *PDGFRB*. A robust method is expected to rank all Kinase family members before non-Kinase matches. In particular, we examine pairing between the Kinase *PRKG1B* and the non-Kinase *NCAM2*, the genes depicted in Figure 2. Neighborhood Correlation exhibits no difficulty separating these pairs. The match with *PRKG1B* scores substantially higher than *NCAM2* (indicated by magenta and green circles, repectively, in Figure 5). In contrast, the BLAST scores for these sequences are indistinguishable, and the PSI-BLAST scores for these sequences are *reversed*: The match to *NCAM2* obtains θ = 3.65*e* ^−40^, while the match to *PRKG1B* is much less significant (θ = 1.26*e* ^−25^). How typical are these examples? As shown in Figure 6, the sequence similarity distributions of FF and FO pairs overlap completely for BLAST and partially for PSI-BLAST. In contrast, the Neighborhood Correlation score distributions for family and non-family matches are largely distinct, with only a limited overlap in the tails of the distributions.

**Figure 7:**
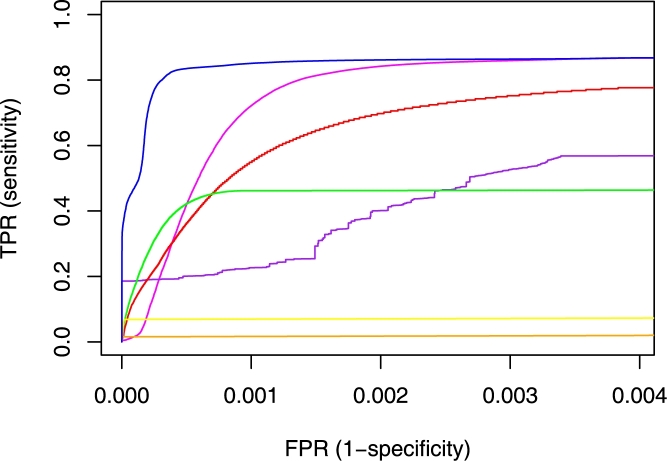
*ROC-100k* curves for the Kinase family for all classification methods tested. *ROC-100k* curves of Neighborhood Correlation (blue), BLAST (red), PSI-BLAST (magenta), DAC (purple) and alignment coverage (*α*≥0.3: green, *α*≥0.6: yellow, *α* ≥0.8: orange).

Neighborhood Correlation also delivers robust performance when sensitivity (*Sn*) and specificity (*Sp*) are considered independently. For example, when matches to the query sequence *PDGFRB* are ranked by Neighborhood Correlation score (Figure 5A), a cutoff of *NC = 0.3* results in three false positives with only ten false negatives. In contrast, a BLAST threshold of *E*<3*e* ^−10^ results in three false positives and 630 false negatives (Figure 5B). The number of false negatives obtained with PSI-BLAST at this specificity is even greater (Figure 5C). More generally, the *ROC-n* curves for the Kinase family in Figure 7 demonstrate that Neighborhood Correlation achieves both higher sensitivity and higher specificity than BLAST, except at very high specificity, and always outperforms PSI-BLAST by both measures. Neighborhood Correlation simultaneously achieves *Sn*≈0.85 and *Sp*≥0.999. At this specificity, *Sn≈*0.7 for PSI-BLAST and *Sn≈*0.55 for BLAST.

While the other methods considered have strengths specific to particular challenges, Neighborhood Correlation delivers the most reliable and consistent performance on large, heterogeneous datasets. Neighborhood Correlation is, therefore, particularly well suited to automated genome-scale analyses, which require that a single classification threshold be suitable for the vast majority of sequence pairs in a genomic dataset. Moreover, Neighborhood Correlation is robust. The distribution of Neighborhood Correlation scores for all sequence pairs in our dataset (Figure S3) has a flat trough ranging from 0.4 to 0.8. Within this range, the prediction quality will be relatively insensitive to the choice of threshold. A putative set of mouse and human homologs imposed by a threshold of *NC*≥0.6 on all sequence pairs in our dataset is available at http://www.neighborhoodcorrelation.org.

### PSI-BLAST

As expected, PSI-BLAST excels at families with low sequence conservation, such as TNF and USP, and generally performs well on single domain families. However, PSI-BLAST falters on complex multidomain families and on sequences with promiscuous domains. PSI-BLAST's average *ROC-100k* scores for both conserved and variable multidomain families are inferior to those of both Neighborhood Correlation and BLAST. This is exemplified by PSI-BLAST's poor performance (Figure 5B) when querying with *PDGFRB*, which has two copies of the highly promiscuous Ig domain. PSI-BLAST's iterative profile construction algorithm incorporates matches to the highly promiscuous Ig domain in the growing alignment, even when a very stringent inclusion threshold (*E*<10^−13^) is used. As a result, unrelated sequences that contain Ig domains match the resulting profile with better scores than Kinases without Ig. PSI-BLAST performs better on the Kinase family as a whole than it does on *PDGFRB* (Table 3) because many Kinases are single domain proteins.

When classification of heterogeneous data is considered, PSI-BLAST's performance is inferior to Neighborhood Correlation on the *ALL* dataset and to both Neighborhood Correlation and BLAST on the *ALL-Kin* dataset. This demonstrates that no single PSI-BLAST cutoff is suitable for all families. Indeed, inspection of PSI-BLAST output on individual queries (data not shown) indicates that PSI-BLAST scores tend to vary widely from family to family. PSI-BLAST introduces a clear tradeoff between sensitivity and generality, to the particular detriment of large-scale studies. Moreover, PSI-BLAST is characterized by greater instability and running time than BLAST or Neighborhood Correlation.

### Domain Architecture Comparison

Domain architecture comparison performs well on single domain families and on multidomain families with conserved domain architectures (e.g., DVL, Notch, Laminin, and WNT). Like PSI-BLAST, DAC can recognize distant homology because domain architectures are recognized by MSA-based models. The performance of DAC on other families is mixed, however, because it faces a number of challenges that do not arise with the other classification methods.

First, all domain architecture comparison methods are substantially restricted by the limitations of domain detection. In our dataset, 12.7% of sequences do not have domain annotations, resulting in low *ROC-100k* scores for many families. This explains why single domain families, such as Tbox, which have identical domain architectures, do not achieve perfect *ROC-100k* scores, contrary to expectations. An additional shortcoming is that domain architecture comparison methods do not capture information in linker sequences or sequence variation within a domain family. Therefore, domain architecture comparison tends to assign the same score to pairs that actually differ in sequence divergence. This explains the long plateaus in the ROC curve for DAC in Figure 7.

A particularly challenging problem for domain architecture comparison is how to effectively distinguish domains that proliferated through gene duplication from promiscuous domains that proliferated through domain shuffling. The number of domain partners, used here, is a typical measure of promiscuity, based on the assumption that this measure reflects the frequency of domain insertion [48]. This measure of promiscuity will inappropriately down-weight a domain that characterizes a family, if the domain happens to be the target of insertions of many other domains. Consider, for example, a sequence with a single domain *A* that sustains repeated duplication, followed by insertion of different domains into the resulting copies, yielding *AB*, *AC*, *AD*, and so on. Domain *A* will have a high promiscuity score, although it is never inserted into new contexts. As a concrete example, the Pkinase domain partners with more than 100 different domains. However, the resulting high promiscuity score may be inappropriate since Pkinase lacks many of the other characteristics of promiscuous domains, such as small size and 1-1 phase [17], and is important in defining the Kinase family. This explains why domain architecture comparison performs poorly on the Kinase family.

### Alignment Coverage

To assess the effectiveness of alignment coverage in eliminating domain-only matches, we compared *ROC-100k* scores for sequence similarity alone and combined with alignment coverage (*α*, see Methods). We considered three alignment coverage thresholds, *α*≥0.3, *α*≥0.6, and *α*≥0.8, that span the range of length cutoffs used in the literature (*e.g.* [32],[34]). The results (Table 4) show that the addition of an alignment coverage criterion does not improve the performance of sequence similarity. For example, a cutoff of *α*≥0.3 reduces the *ROC-100k* score by 25% in the *ALL* dataset and 23% in the *ALL-Kin* dataset. When families are considered individually, a cutoff of *α*≥0.3 decreases the *ROC-100k* score by at least 10% in one-third of the families. Increasing the cutoff to *α*≥0.6 or *α*≥0.8 does not increase performance in any family. Note that although the *ROC-100k* score for KIR when *α*≥0.6 is higher than the score for sequence similarity alone, this difference is not significant (*p = 0.69*).

**Table 4:** *ROC-100k* scores for BLAST alone, and combined with alignment coverage at thresholds of *α*≥0.3, *α*≥0.6, and *α*≥0.8.

	BLAST	*α*≥0.3	*α*≥0.6	*α*≥0.8
*ALL*	**0.5838**	0.4295	0.0784	0.0236
*ALL-Kin*	**0.7505**	0.5756	0.2902	0.1747
Single domain families				
ACSL	**1.0000**	**1.0000**	**1.0000**	**1.0000**
FGF	**0.9920**	0.9757	0.6002	0.1403
FOX	**0.9996**	0.3172	0.0635	0.0310
Tbox	**1.0000**	0.9740	0.1883	0.1136
TNF	**0.3631**	0.3588	0.2090	0.0814
USP	**0.8666**	0.3312	0.1230	0.0609
WNT	**1.0000**	**1.0000**	**1.0000**	**1.0000**
Mean	**0.8888**	0.7081	0.4549	0.3467
Multidomain families: conserved architecture				
DVL	**1.0000**	**1.0000**	0.7755	0.2653
GATA	**1.0000**	0.8679	0.4097	0.3125
Notch	**1.0000**	**1.0000**	**1.0000**	**1.0000**
KIR	0.9971	0.9971	**0.9973**	0.7597
TRAF	**1.0000**	**1.0000**	0.8401	0.8403
Mean	**0.9994**	0.9730	0.8045	0.6356
Multidomain families: variable architecture				
ADAM	**0.9830**	0.9372	0.8772	0.4744
Kinase	**0.6164**	0.4384	0.0704	0.0176
Kinesin	**0.9806**	0.7644	0.1582	0.0842
Laminin	**0.9245**	0.5681	0.2836	0.1640
Myosin	**0.9870**	0.8804	0.4482	0.2682
PDE	**0.7565**	0.7311	0.1960	0.1424
SEMA	0.9983	**0.9998**	0.6409	0.3493
TNFR	**0.5607**	0.3927	0.0703	0.0453
Mean	**0.8509**	0.7140	0.3431	0.1932

Alignment coverage is based on the assumption that non-homologous pairs have shorter regions of similarity than homologous pairs, yet Table 4 suggests this is not universally true. To assess the extent to which the region of similarity in homologous pairs extends over the bulk of their length, we calculated Precision and Recall (see Methods) for *α*≥0.3, *α*≥0.6, and *α*≥0.8. The results, shown in Tables 5 and Table S1, suggest that full length alignments are not a characteristic property of homologous families, at least in our dataset. In the *ALL-Kin* dataset, a cutoff of *α*≥0.3 eliminates 40% of true positives, specifying *α*≥0.6 eliminates 70% of true positives, and *α*≥0.8 eliminates 83% true positives. The loss in Recall is even more extreme in the *ALL* dataset.

**Table 5:** Precision and recall for predictions using optimal and combined alignments.

	*α*≥0.3		*α*≥0.6		*α*≥0.8	
	Precision	Recall	Precision	Recall	Precision	Recall
Optimal alignment						
*ALL*	0.8810	0.4675	0.9556	0.0772	0.9893	0.0217
*ALL-Kin*	0.3775	0.6072	0.7853	0.2904	0.9758	0.1732
Combined alignments						
*ALL*	0.8776	0.4777	0.9549	0.0787	0.9889	0.0220
*ALL-Kin*	0.3807	0.6528	0.7861	0.2999	0.9750	0.1771

**Figure 8:**
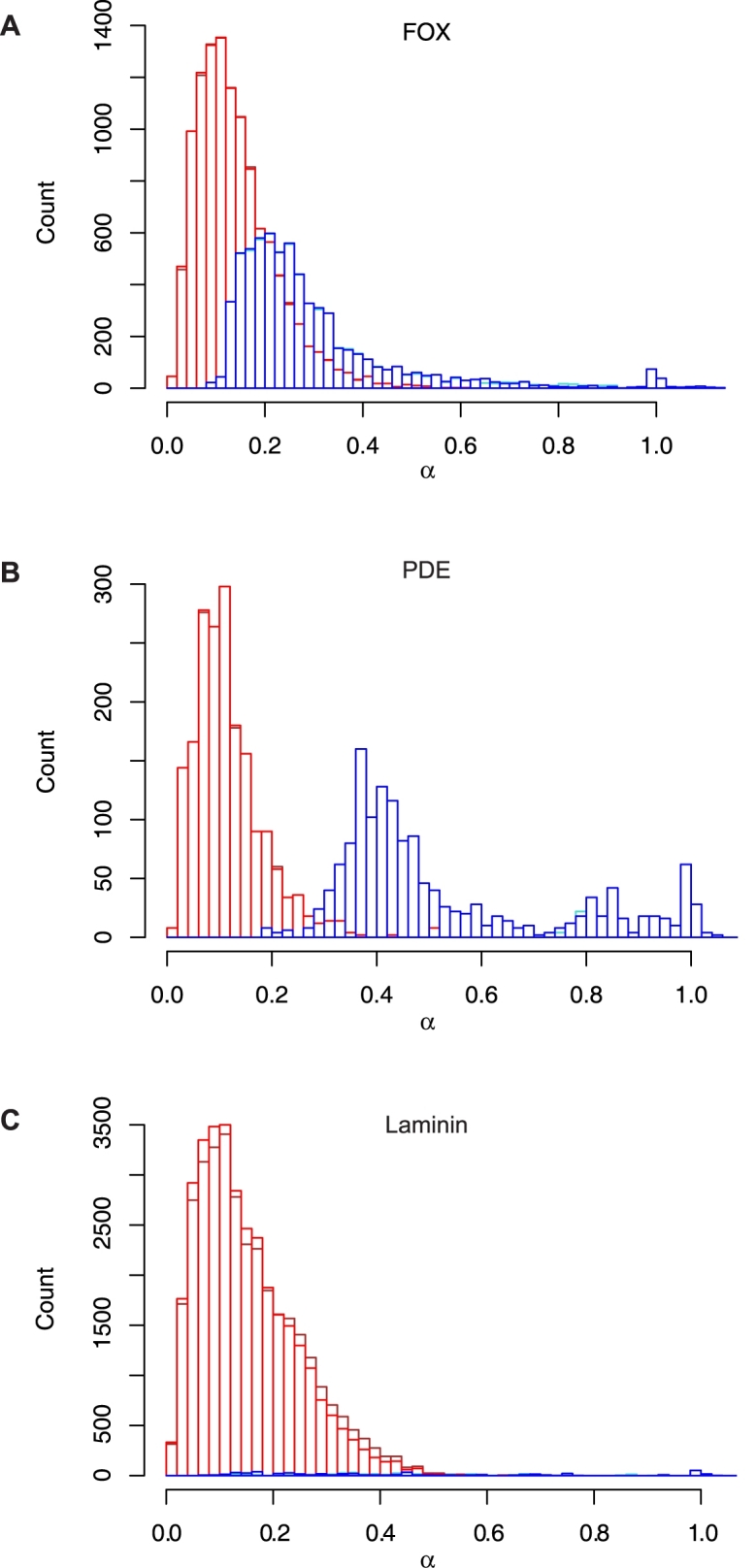
Alignment coverage distributions for representative families. Histograms calculated with the optimal alignment length only (FF: blue, FO: red) and with combined non-conflicting alignments (FF: turquoise, FO: brown) (A) FOX, (B) PDE, and (C) Laminin.

To better understand these results, we plotted histograms of *α* for individual families (Figures 8, S4). While some families do have long regions of similarity, long conserved regions are not a persistent characteristic of most families in our dataset. Several different trends in domain organization can cause this. Some families are characterized by a short, conserved domain, such as the DNA binding domain in the FOX family, and little conservation elsewhere (Figure 8A). Multidomain families exhibit a range of alignment lengths for a variety of reasons. In families characterized by a single defining domain partnered with a variety of auxiliary domains, alignment lengths depend upon the number of domains a given pair has in common. For example, the histogram for the PDE family (Figure 8B) has a small peak near *α* = 1.0, corresponding to pairs with identical domain architectures, and a much larger peak between *α* = 0.2 and *α* = 0.7 that represents pairs of family members with different auxiliary domains. Families can also demonstrate wide variation in due to differences in copy number (*e.g.*, Laminin, Figure 8C). Finally, a broad *α* distribution can be caused by variation in sequence length within the family. Even when the length of the conserved region is constant, alignment coverage, expressed as a fraction of total length, may vary widely, confounding homology prediction methods based upon alignment coverage.

Given the widespread use of alignment coverage criteria, we were surprised by this poor performance. We examined the possibility that our failure to observe a consistent pattern of long alignments was due to the fact that we considered the length of the optimal alignment, only. To investigate whether including sub-optimal alignments would result in different conclusions, we implemented a simple heuristic (see Methods) that identifies and combines a consistent set of high-scoring local alignments; i.e., alignments that appear in the same order in both sequences and do not overlap. Surprisingly, including suboptimal alignments in the alignment coverage calculation has little impact on our results. The distributions of the combined alignment lengths, shown in turquoise and brown in Figures 8 and S4, differ little from the distribution of optimal alignment length distributions (shown in blue and red). Nor do the values of Precision and Recall obtained with combined alignments differ greatly from those obtained with the optimal alignment (see Table 5 and Table S2). In summary, analysis with combined alignments confirms that full length similarity is not a general characteristic of homologous families.

## Discussion

Protein modularity allows the evolution of diverse function through combinatorial rearrangement of functional building blocks. This versatile evolutionary mechanism played a transformative role in key evolutionary transitions, including the emergence of multicellular animals and the vertebrate immune system. Identification of multidomain homologs is essential to studying the evolution of modular families, as well as to many genomic applications that exploit evolutionary information.

Two obstacles have impeded research on multidomain homology: the absence of formal models and a lack of curated datasets of multidomain homologs for evaluation of proposed methods. In the current paper, we offer preliminary solutions to both problems: We propose an evolutionary model and an associated definition of homology suitable for multidomain proteins. We further provide a curated test set of homologous mouse and human sequence pairs from twenty well-studied families for which there is unambiguous evidence that member sequences are derived from a common ancestor. Our benchmark encompasses various challenges for homology identification methods, including both conserved and variable multidomain architectures, promiscuous domains, single domain families with short regions of conservation, and families with weak sequence conservation. It differs from other available benchmarks in that it seeks to represent evolutionary, rather than structural (e.g., SCOP [38]) or functional (e.g., GO [59]) information. This benchmark is available to the community through the Neighborhood Correlation website.

Using our curated benchmark, we demonstrate that the most widely used homology identification methods, BLAST, PSI-BLAST, domain architecture comparison, and alignment coverage, all face serious limitations in their ability to recognize multidomain homologs. In response, we introduce Neighborhood Correlation, a method that uses a fundamentally different approach to homology identification by deriving evolutionary signal from the local structure of the sequence similarity network. Following a discussion of our model within the historical framework of models of homology, we place our results in the perspective of similar problems and approaches. We discuss Neighborhood Correlation in relation to other evolutionary classifications, the needs of genomic applications and multiple sequence alignment methods, and conclude by reviewing the potential of networks in molecular evolution.

### Model

Although models of gene family evolution have been proposed and debated for more than three decades [18], models of multidomain evolution are in their infancy. Gene homology is a yes/no question: genes either share common ancestry or they do not. With this in mind, Fitch [42] argued that when subsequences of genes have distinct evolutionary histories, it is not possible to determine gene homology. Rost and colleagues [45],[63] further proposed that “dissecting proteins into structural domain-like fragments” [45] is the only reasonable way to study relationships in such proteins. We suggest an alternative: By considering the genomic context of genes that encode multidomain proteins, it is possible to define homology for multidomain sequences without violating the tenet that homology is an indivisible property.

We propose a model of multidomain evolution in which the set of events by which sequences diverge is expanded to include domain insertion and deletion as well as mutation. Recent evidence from studies of young genes [50]–[53], as well as indirect evidence of sequence shuffling [17],[24],[49],[55],[56], suggests that our model is consistent with a significant fraction of metazoan multidomain families. This model permits discrimination between genes related by vertical descent and those related by domain insertion alone, which is the basis for our definition of multidomain homology. This in turn enlarges the scope of inquiry from domain family homology to gene family homology, providing a broader context in which to study the evolutionary processes by which modular families are formed. Our model does not describe families that evolved through other domain shuffling processes such as gene fission, the fusion of adjacent genes resulting from read-through errors, or *de novo* formation of novel architectures through independent insertions in intergenic regions. Extending the model to capture a broader range of domain shuffling scenarios and testing it on other datasets and applications are important directions for future work.

### Comparison with Other Evolutionary Classifications

**Figure 9:**
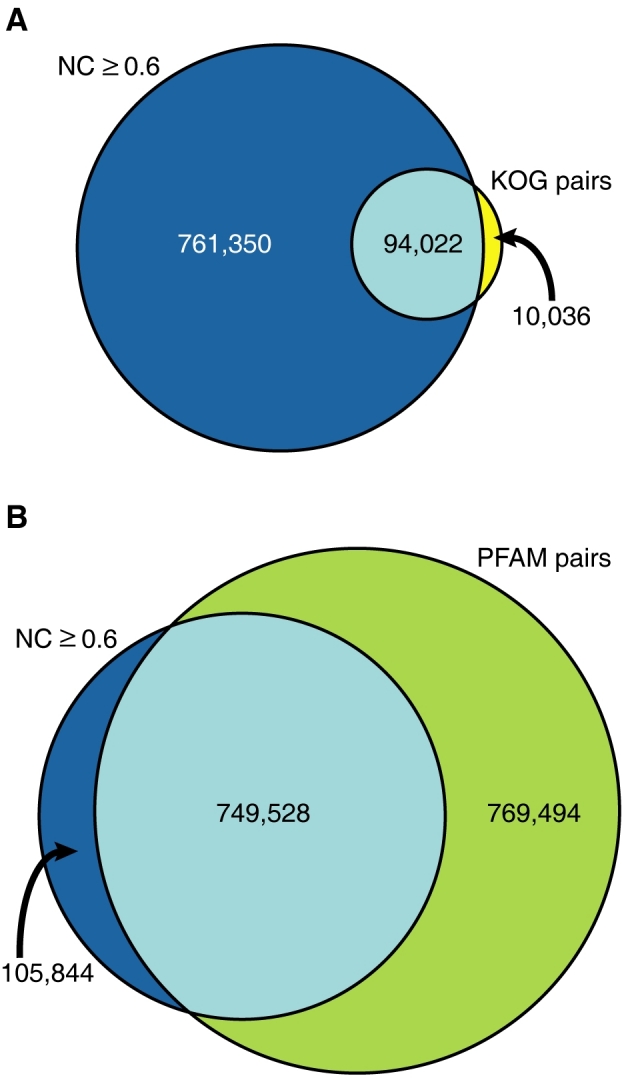
Comparison of Neighborhood Correlation with other classifications. (A) Venn diagram representing pairs with *NC*≥0.6 that share a KOG annotation (turquoise), pairs with *NC*≥0.6 that do not share a KOG annotation (blue), and pairs with *NC*<0.6 that share a KOG annotation (yellow). (B) Pairs with *NC*≥0.6 that share a Pfam domain (turquoise), pairs with *NC*≥0.6 that do not share a Pfam domain (blue), and pairs with *NC*<0.6 that share a Pfam domain (green).

Evidence supporting the validity of our model can be obtained by comparing Neighborhood Correlation with related classifications, such as orthology and domain homology. The success of Neighborhood Correlation in recapitulating homologous relationships in our benchmark empirically supports Neighborhood Correlation as a predictor of homologous genes; that is, sequences derived from a common ancestor by vertical descent, whether by duplication or speciation. Since orthologs, sequences that diverged by speciation in their most recent common ancestor, are by definition homologs, our model predicts that known mouse and human orthologs will have high Neighborhood Correlation scores. To test this prediction, we compared Neighborhood Correlation with KOGs [40]. As expected, 90% of sequences in our dataset with the same KOG annotation have a Neighborhood Correlation Score greater than 0.6 (Figure 9A). However, only 12% of pairs with *NC*≥0.6 share the same KOG annotation. This is consistent with the observation that gene homology is a necessary but not sufficient condition to establish orthology.

Domain homology, on the other hand, is a less stringent criterion than gene homology. Homologous genes, by definition, share at least one homologous domain. Of pairs with Neighborhood Correlation scores above 0.6, 88% of pairs share at least one Pfam [41] code (Figure 9B), consistent with the assertion that gene homology is a more stringent requirement than domain homology. That the remaining 12% do not share a domain is primarily due to missing annotations. Recall that 12.7% of sequences in our dataset do not contain a recognizable Pfam domain.

Since only some sequences that share a domain are encoded by homologous genes, our model predicts that a significant fraction of sequence pairs that share homologous domains will not have high Neighborhood Correlation scores. In fact, with *NC*≥0.6, only half of sequence pairs in our dataset share a Pfam domain. These results are consistent with the expectation that gene homology is a less restrictive condition than orthology but more restrictive than domain homology. This analysis provides additional evidence, independent of our curated dataset, that Neighborhood Correlation can predict homologous genes according to our model.

### Empirical Evaluation for Genome-Scale Analyses

Insight into the evolutionary processes responsible for the development of novel function are of greatest value when considered in the context of entire genomes. To accommodate studies of such scale, a method must be suitable for robust, automated analyses. For the current application, this requires speed, ease of use, and consistent behavior across varied domain architectures.

Neighborhood Correlation displays excellent performance across an array of families with a range of sequence patterns and evolutionary histories. Neighborhood Correlation is able to correctly classify complex families, while maintaining accuracy on simpler families. Further, it displays a classification threshold that is robust with respect to family, yielding good performance on individual families as well as on aggregate datasets in which families may not be known or readily discernible. Since Neighborhood Correlation can be computed easily with existing computing resources and data stores, it is easy to add to a computational workflow. These qualities demonstrate that Neighborhood Correlation is well suited to large-scale genomic analysis.

Empirical evaluation of existing homology detection methods revealed limitations in their applicability, often contrary to common expectations. Meticulous tests of BLAST and PSI-BLAST performance have been carried out on well-characterized datasets [58],[62],[64], but, to our knowledge, performance on multidomain proteins with promiscuous domains and low complexity regions has not been considered empirically. Our tests on datasets with multidomain sequences, promiscuous domains, and low complexity regions show that while BLAST represents an attractive balance between speed and accuracy on conserved, single-domain families, additional screening is needed for correct multidomain classification.

Since Huynen and Bork [27] proposed that alignment length could be used to reduce false positives in ortholog prediction, the practice of pre-screening using an alignment coverage criterion has become widespread in genomic analyses [28]–[37]. To determine the effectiveness of this approach, we investigated the two hypotheses underlying the use of alignment coverage:

1. The region of similarity in homologous sequence pairs covers a significant fraction of their length.
2. The fraction of sequence length covered by the aligned region is typically larger in homologous pairs than in unrelated sequence pairs that share an inserted domain.

Surprisingly, the imposition of an alignment coverage requirement, in addition to sequence similarity, actually *decreased* the accuracy of homology identification, suggesting that the above hypotheses are not generally true. To our knowledge, this is the first rigorous evaluation of alignment coverage.

Our study suggests that PSI-BLAST, while first-rate for detecting remote homology, is ill-suited to large scale automated analyses on datasets with complex multidomain architectures, promiscuous domains, and low complexity sequences due to its running time, instability, and family dependent score thresholds. The same iterative strategy that confers PSI-BLAST's increased sensitivity leads to a lack of robust behavior when PSI-BLAST is run in an automated manner. Even at extremely stringent inclusion thresholds, false positives are incorporated in during model construction when the query sequence contains promiscuous domains or low complexity regions. Once a false positive is included, PSI-BLAST rapidly degrades the MSA used in subsequent iterations, leading to both incorrect results and excessively long running times. PSI-BLAST required 208 CPU days for our dataset, a 300-fold increase in time over basic BLAST. This slowdown is associated with the large fraction of promiscuous, multidomain, and low complexity sequences in our dataset. When PSI-BLAST is used interactively, the user can eliminate potentially troublesome matches by inspection; however, human intervention is not possible for genome-scale studies. The additional computational cost of calculating Neighborhood Correlation scores once a BLAST search has been performed is negligible. Though PSI-BLAST does offer accuracy improvement over Neighborhood Correlation on families with conserved domain architectures, these issues suggest that PSI-BLAST is impractical for this or larger genomic studies.

Domain architecture comparison performs well on families with low sequence conservation due to the discrimintatory power of multiple alignment based domain models, yet our empirical evaluation of DAC reveals several areas for improvement. Domain architecture comparison can be compromised by faulty or incomplete domain annotation. Failure to capture sequence variation within domain and linker sequences results in an inability to resolve family substructure. A model of promiscuity that better captures domain mobility is needed to correctly classify families defined by a single domain with many partners. Because the sequence similarity network reflects both domain architecture and sequence variation, Neighborhood Correlation avoids many of these difficulties, including unresolved family substructure and sensitivity to domain annotation. Neighborhood Correlation captures modular organization on a range of scales, including sequence motifs as well as structural domains, regardless of whether these subunits are encoded in a database. In addition, Neighborhood Correlation's success on kinase classification, relative to DAC, suggests that it may be possible to derive accurate promiscuity measures from the network.

### Neighborhood Correlation and Multiple Sequence Alignment

Neighborhood Correlation differs fundamentally in both goals and approach from Position Specific Scoring Matrices, Profile hidden Markov models, PSI-BLAST, and similar methods that exploit multiple alignments to detect distant homology. MSA-based approaches are not suitable for detecting multidomain homologs with varied architectures. These rely upon full length alignments that are not possible with multidomain sequences. The objective of multiple alignment methods is to identify related sequence motifs when the signal to noise ratio is low. In contrast, the goal of Neighborhood Correlation is to identify homologs that have sustained domain insertions and deletions since their divergence.

Neighborhood Correlation also differs from methods based on multiple alignment in its computational approach. Although both approaches derive information from neighboring sequences, only Neighborhood Correlation exploits the topology of the network. MSA-based methods synthesize a model from a set of neighbors in the sequence similarity network and then use the resulting composite model in pairwise comparisons. Such models reflect aggregate properties of the network neighborhood, but not the underlying topological structure of the network. In contrast, Neighborhood Correlation compares the edge weights for each pair of shared neighbors separately, capturing not only neighborhood membership, but also specific information about how individual sequences in the neighborhood are related. Finally, Neighborhood Correlation derives information from neighborhood difference as well as from neighborhood similarity, taking advantage of the fact that sequences that match one member of the pair and not the other are informative.

### Evolutionary Information in Similarity Networks

Neighborhood Correlation complements a recent set of studies relating multidomain evolution to the *global* topological properties of the domain similarity network [65]–[69]. Unlike these methods we focus on *local* network structure as evidence of the evolutionary history of specific sequence pairs and families. In an early use of local network structure, Koonin and colleagues [40] argued that orthologous groups correspond to cliques in the sequence similarity network. In a similar vein, Przytycka and colleagues [70],[71] used a different aspect of local structure (chordality) to test whether domain insertion and intron acquisition are evolving in a parsimonious manner in a given family. In a recent study of protein families in prokaryotes, Medini *et al.* [72] consider local network structure, but do not relate it to evolutionary processes. In their study, they developed a scoring system based on sets of nearest neighbors in an unweighted network and used these pairwise scores to identify core sets of proteins associated with secretion systems in prokaryotes.

Neighborhood Correlation links local network structure to both domain architecture and evolutionary process. The similarities and differences in domain architecture are reflected in the neighborhoods of adjacent sequences. The number and weights of edges in the shared neighborhood is influenced by the number and conservation of their shared domains. Their unique neighborhoods are similarly influenced by their unique domains. The Neighborhood Correlation score, therefore, is an implicit measure of both sequence similarity and domain architecture comparison.

The history of gene duplication and domain insertion in gene family evolution is also recorded in network topology. Neighborhood Correlation is able to elucidate multidomain homology because it can decipher the traces of this history in the network. In particular, Neighborhood Correlation relies on the hypothesis that the neighborhoods of genes related through duplication are more similar to each other than the neighborhoods of genes related through domain insertion. This hypothesis in turn assumes that

1. gene duplication occurs more frequently than domain insertion, and
2. the promiscuity and sequence conservation of domain superfamilies are inversely related.

There is concrete evidence to support the latter assertion as indicated by the negative correlation between the promiscuity and sequence identity of Pfam domains, discussed in Results. We are not aware of any studies predicting the relative rates of gene duplication and domain insertion. However, the success of Neighborhood Correlation in classifying multidomain homologs provides indirect evidence that the assertion is true, at least in the dataset studied here. If, contrary to this hypothesis, domain insertions occurred as or more frequently than gene duplications, the Neighborhood Correlation scores of multidomain homologs would not be distinctly higher than those of domain-only matches.

More generally, the success of Neighborhood Correlation has demonstrated that information about the interplay of the processes of gene duplication, domain shuffling, and sequence divergence lies hidden in the local structure of the sequence similarity network. This success suggests that mining network structures is a promising direction for extending bioinformatics methodology, as well as for asking basic questions about evolutionary processes. For example, it has been argued that the increased complexity of multidomain families in metazoans is directly related to the advent of multicellular animals. Multicellularity has evolved several times ([73] and work cited therein). In each case, Nature has had to evolve novel solutions to the problems of coordinated cellular communication and control. It is an intriguing question whether the same patterns of gene duplication and domain insertion that prompted the evolution of metazoan signal transduction families also dominate in other lineages. Future work will determine whether we can further exploit local organization of the sequence similarity network to investigate such questions.

## Methods

### Data

We extracted all complete mouse and human protein sequences from SwissProt Version 50.9 [74], yielding 11,553 mouse protein sequences and 14,644 human protein sequences. Sequence fragments were excluded from this set of sequences by rejecting sequences annotated with a description field containing “(fragment”. We chose SwissProt, a high quality, curated protein sequence database, as opposed to GenBank, which would have resulted in a larger, but less reliable, dataset. KOG annotations were obtained from the Clusters of Orthologous Groups database [40], available from ftp://ftp.ncbi.nih.gov/pub/COG/KOG/. KOG annotations were mapped to SwissProt identifiers by exact matching of KOG FASTA protein sequences with those in SwissProt.

The analysis was carried out on the combined set of mouse and human sequences. In a preliminary study, we compared the performance of Neighborhood Correlation on a smaller, combined set of mouse and human sequences with its performance on separate sets of mouse and human sequences [75] to determine whether Neighborhood Correlation performs differently on comparisons within and across genomes. The mouse-only and human-only data test the ability to classify paralogs within a single mammalian species, as opposed to the combination of orthologs and paralogs seen in the combined dataset. The basic trends in the mouse-only and human-only datasets were the same as the combined dataset for all tests performed. This suggests that Neighborhood Correlation performance is not highly sensitive to the degree of sequence divergence, since paralogous and orthologous sequences in these species exhibit different patterns of divergence.

### Family Identification

For each family, we derived a list of designated gene symbols, Pfam [41] and/or InterPro [76] codes from publications by family experts, and reports from the Human Genome Nomenclature Committee (http://www.gene.ucl.ac.uk/nomenclature/genefamily.html). These lists were used to generate a preliminary roster for each family, then confirmed by referring to recent analyses of gene family evolution in the literature. A detailed account of the curation procedure for each family with specific identification criteria and references is given in Text S1. SwissProt accession numbers for all sequences in the twenty families are provided in Dataset S1.

### Sequence Comparison

We conducted all-against-all BLAST (Version 2.2.15) [61] and PSI-BLAST (Version 2.2.16) [58] searches for the sequences in our dataset, using the BLOSUM 62 matrix, an affine gap penalty of −*(11+k)* for a gap of length *k*, and low complexity filtering. For both searches, the size of the search space was set to *Y = n* ^2^ and the significance threshold to *E = 10N*, where *n* is the size of the database in residues and *N* is the number of sequences in the dataset.

The combined dataset has *N = *26,197 sequences, 11,553 mouse and 14,644 human sequences, corresponding to a total of *n = *14,073,417 residues. For PSI-BLAST, four passes were executed with an inclusion threshold of *E*<10^−13^ for inclusion in the multiple alignment used to search in the next pass. Although this cutoff is much more stringent than the default, we found it essential to obtain correct results with sequences containing low complexity regions. Less stringent thresholds resulted in the inclusion of unrelated sequences in the intermediate PSSM. Asymmetries (i.e., *E*(*x*,*y*)≠*E*(*y*,*x*)) that occur due to low complexity filtering [77], which is applied only to the query sequence but not to database sequences, were corrected by assigning the better of the two values to both matrix entries. The resulting dataset had 4,864,226 significant BLAST pairs and 10,854,626 significant PSI-BLAST pairs.

The parameter values used in this study embody the view that an all-against-all BLAST search is a single experiment. This approach is roughly equivalent to conducting *N* single query BLAST searches with *E = *10 and *Y = m_x_ n*, where *m_x_* is the length of query sequence *x*. Treating the all-against-all BLAST comparison as a single experiment results in symmetric E-values in the absence of low complexity filtering. We define θ(*x*,*y*) = *E*(*x*,*y*)/10*N* to be the expected number of chance hits per sequence in the dataset with a score equivalent to, or better than, that of the alignment of query sequence *x* with matching sequence *y*. The significance threshold of *E = *10*N* corresponds to θ = 10 chance hits per sequence, in expectation.

### Neighborhood Correlation Score Calculation

We calculated the Neighborhood Correlation scores for all sequence pairs in our dataset from Equation 1 using the similarity score,
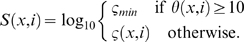
(2)where ς(*x*,*i*) is the normalized bit score [58] of the alignment of *x* and *i* and ς*_min_*(*x*,*i*) = log_2_(*n* ^2^/10*N*)*0.95 = 28.019, which is 5% less than the bit score corresponding to θ = 10 for a dataset of the size used in this study.

The effectiveness of Neighborhood Correlation depends strongly on how the similarity score, *S(x, i)*, is defined. We considered three measures of similarity: *S*(*x*,*i*) = log ς(*x*,*i*), *S*(*x*,*i*) = ς(*x*,*i*) and an unweighted comparison of neighborhood membership defined as *S*(*x*,*i*) = 1 if there is a significant match between *x* and *i*, and zero otherwise. Although the other two measures performed well on some families, only *S*(*x*,*i*) = log ς(*x*,*i*) gave consistent, good performance on a wide range of families. This suggests two factors that may be important to Neighborhood Correlation performance. First, the relatively poor performance of the unweighted score indicates that it is necessary to capture differences in the degree of similarity to sequences in the neighborhood to capture complete evolutionary information. Second, the improved performance obtained with *S*(*x*,*i*) = log ς(*x*,*i*) can be understood by recalling that the correlation coefficient captures only linear associations. The use of the logarithm compresses the range of ς(.,.), resulting in scores that more closely approximate linearity.

The choice of ς*_min_*, the score assigned to pairs without significant similarity, may influence Neighborhood Correlation performance in homology identification. We experimented with values of ς*_min_* corresponding to significance thresholds ranging over two orders of magnitude. The results (data not shown) suggest that varying ς*_min_* has little impact on Neighborhood Correlation.

### Promiscuity and Sequence Identity

Promiscuity refers to the tendency of domains to be inserted into many different contexts. Typically, promiscuity of a domain is defined as the number of distinct partners associated with it, where two domains are *partners* if they co-occur in at least one sequence [3]. We obtained the set of Pfam codes associated with all sequences in our dataset from the SwissProt database. For each Pfam domain, we determined the number of distinct Pfam codes that co-occur with it in any of the 26,197 sequences in our dataset.

We further obtained percent sequence identity for each Pfam identifier from the Pfam website. The Spearman ranked correlation coefficient of domain promiscuity and sequence identity was calculated to evaluate whether promiscuity and sequence identity were related.

### Domain Architecture Comparison

We conducted an all-against-all domain architecture comparison using the Pfam identifiers provided by SwissProt. Similarity of each pair of sequences, *x* and *y* were calculated as follows:
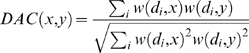
(3)where *w*(*d_i_*,*x*) is the weight of domain *d_i_* in sequence *x*. Domains are assigned weights inversely proportional to their promiscuity. Promiscuous domains may occur in many unrelated sequences, and so are less useful than relatively rare domains in determining homology. The weight of a domain not contained in a given sequence is zero. As a result, pairs of sequences which share no domains are assigned a similarity of zero. This domain architecture comparison function corrects for the bias of proteins with many domains. Proteins with numerous domains have an elevated probability of sharing a domain with other proteins. Of the 21 domain architecture comparison methods we evaluated in a previous study [23], this was shown to have the best performance.

### Alignment Coverage

For every pair of sequences, *x* and *y*, with significant similarity, we calculated the alignment coverage, defined as *α*(*x*,*y*) = 2*l_a_*/(*l_x_*+*l_y_*), where *l_x_* and *l_y_* are the length of sequences *x* and *y*, and *l_a_* is the length of the optimal local alignment, define to be the number of columns needed to represent it; that is, it includes gapped positions. The length of the optimal alignment between query *x* and match *y* will not, in general, be the same as the length of the optimal alignment between query *y* and match *x*. We forced the alignment coverage to be symmetric by setting both *α*(*x*,*y*) and *α*(*y*,*x*) to the maximum of the two values.

By considering only the optimal alignment, we risk underestimating the extent of similarity between homologous sequences. To take suboptimal alignments into account, we used a simple heuristic method for selecting a set of high-scoring local alignments that do not conflict. Two alignments conflict if they overlap or do not appear in the same order in both sequences (see Text S1).

### Validation

Classifier performance was evaluated using Receiver Operating Characteristic (*ROC*), which captures the tradeoff between sensitivity (*S_n_*) and specificity (*S_p_*) as a function of the classifier threshold. A *ROC* curve is a plot of *S_n_* as a function of 1−*S_p_*, where *S_n_* = *TP*/(*TP*+*FN*) and *S_p_* = *TN*/(*TN*+*FP*). *TP*, *FP*, *TN*, and *FN* refer to the number of True Positives, False Positives, True Negatives, and False Negatives, respectively. In the context of our test, *TP* is the number of sequence pairs that have common ancestry and have been correctly identified by the classifier. *FP* represents the number of pairs that are classified as homologs, but are not family pairs. *TN* and *FN* refer to the number of non-homologous pairs that are correctly ruled out and incorrectly included, respectively.

The area under the *ROC* curve provides a single measure of classification accuracy, corresponding to the fraction of correctly classified entities given the best possible choice of threshold. We used the *ROC-n* score, defined to be the area under the *ROC* curve truncated after the first *n* false positives or
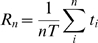
(4)where *t_i_* is the number of FF pairs observed before the *i^th^* FO pair and *T* is the total number of FF pairs in the dataset. When the number of negative examples far exceeds the number of positive examples, as is the case here, the *ROC* score approaches one, resulting in an unjustifiably optimistic assessment of classifier performance. *R_n_* is a more sensitive figure of merit than the untruncated *ROC* score in this case [78]. We selected *n* = 100*k*, where *k* is the number of FF pairs. This is equivalent to 100 false positives per query. We found that 100*k* was sufficiently large so that few FF pairs were missed in most tests but not so large so as to obscure the differences in performance between classifiers.

The statistical significance of the difference between the *ROC-n* scores obtained by Neighborhood Correlation and sequence similarity was estimated using p-values calculated using the method described in Schaffer *et al.* [62]. This method tests the null hypothesis that the difference in *ROC-n* scores is due the sampling process used to obtain the test data. Rejection of the null hypothesis indicates that the difference in *ROC-n* scores represents a true difference in the performance of the classifiers.

Precision and Recall are also used for evaluation. In the context of our test, Recall denotes the fraction of homologous pairs retrieved and is equivalent to sensitivity. Precision refers to the fraction of protein pairs retrieved that are actually homologous pairs.

### Supporting Information on Our Website

http://www.neighborhoodcorrelation.org

- FASTA sequences for all 26,197 human and mouse sequences used in our study.
- The complete set of sequences in each family of our manually curated benchmark.
- A list of Pfam annotations for each sequence used in our study.
- The complete set of NC scores for all sequence pairs.
- Novel predictions of mouse and human homologs using our method (*NC*≥0.6).

### Accession Numbers

The accession numbers used in this paper are from Swiss Prot (http://www.ebi.ac.uk/swissprot): human *PDGFRG* (P09619), human *PRKG1B* (P14619), and mouse *NCAM2* (O35136). Accession numbers for all 1577 sequences in the twenty families in our benchmark are given in Dataset S1.

## Supporting Information

Figure S1 ROC-100k curves for all families. ROC-100k curves of Neighborhood Correlation (blue), PSI-BLAST (magenta), DAC (purple), and BLAST sequence similarity with alignment coverage thresholds of *α*≥0.0 (red), *α*≥0.3 (green), *α*≥0.6 (yellow), and *α*≥0.8 (orange) for all families.
(0.15 MB PDF)

Figure S2 Distributions of BLAST and NC scores for all families. (FF: blue, FO: red).
(0.04 MB PDF)

Figure S3 Distribution of Neighborhood Correlation scores for all sequence pairs.
(0.00 MB PDF)

Figure S4 Distributions of alignment coverage for all families. Distributions of alignment coverage calculated with the optimal alignment length only (FF: blue, FO: red) and with combined non-conflicting alignments (FF: turquoise, FO: brown) for all families.
(0.03 MB PDF)

Table S1 Precision and Recall for predictions using simple alignment coverage thresholds of 0.3, 0.6, and 0.8 for all families.
(0.07 MB DOC)

Table S2 Precision and recall for predictions using combined alignment coverage thresholds of 0.3, 0.6, and 0.8 for all families.
(0.07 MB DOC)

Dataset S1 Curated Benchmark.
(0.02 MB TDS)

Text S1 Supporting Text.
(0.09 MB DOC)
