# Supplementary material for: Sequence Similarity Network Reveals Common Ancestry of Multidomain Proteins
Source: PLoS Comput Biol. 2008 May 16;4(5):e1000063. doi: 10.1371/journal.pcbi.1000063 (PMC2377100; doi:10.1371/journal.pcbi.1000063)
Supplement: Figure S1 — ROC-100k curves for all families. ROC-100k curves of Neighborhood Correlation (blue), PSI-BLAST (magenta), DAC (purple), and BLAST sequence similarity with alignment coverage thresholds of α≥0.0 (red), α≥0.3 (green), α≥0.6 (yellow), and α≥0.8 (orange) for all families. (0.15 MB PDF) [file pcbi.1000063.s001.pdf]

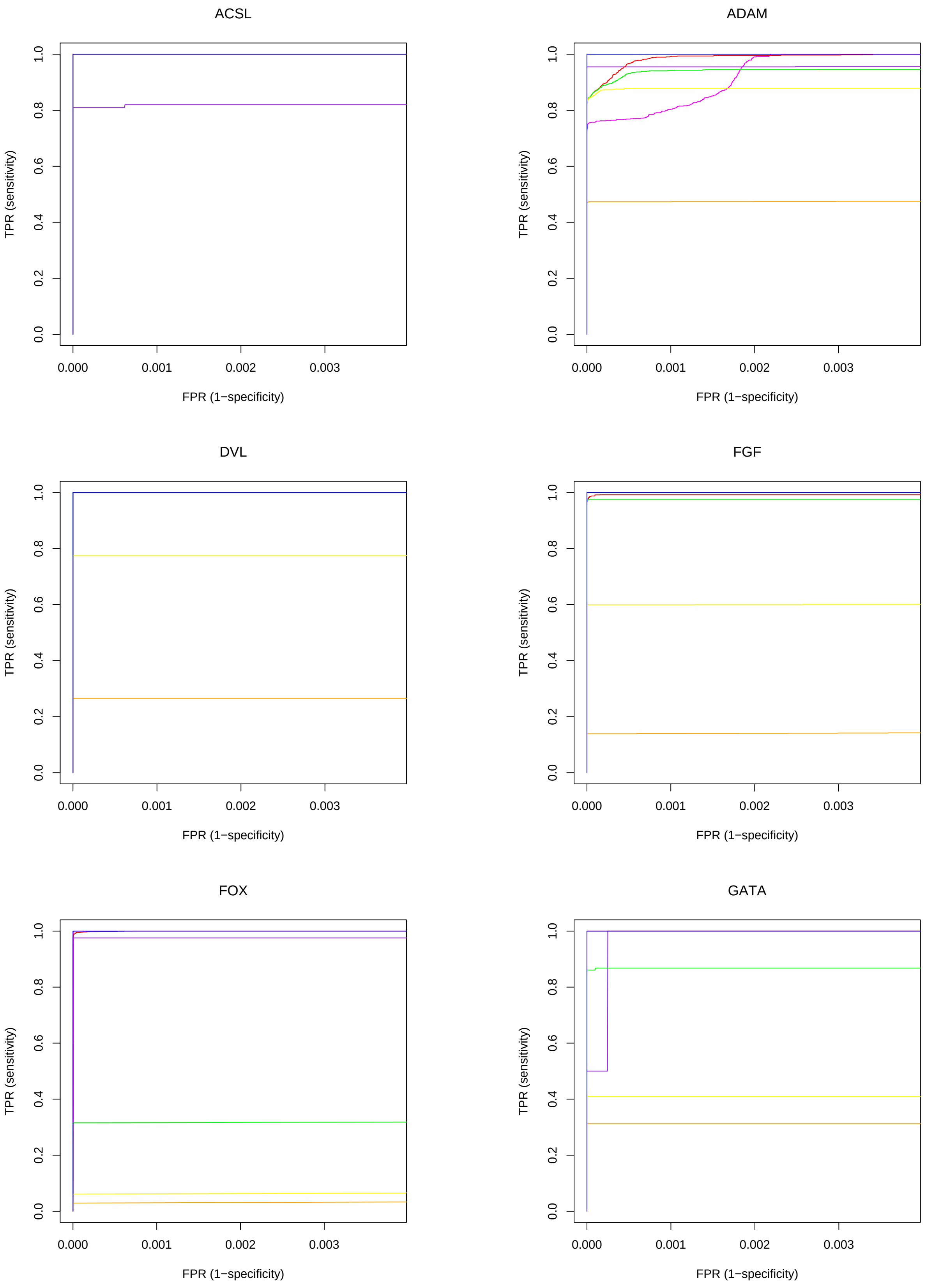

ACSL
ADAM
DVL
FGF
FOX
GATA
TPR (sensitivity)
FPR (1−specificity)
0.0
0.2
0.4
0.6
0.8
1.0
0.000
0.001
0.002
0.003

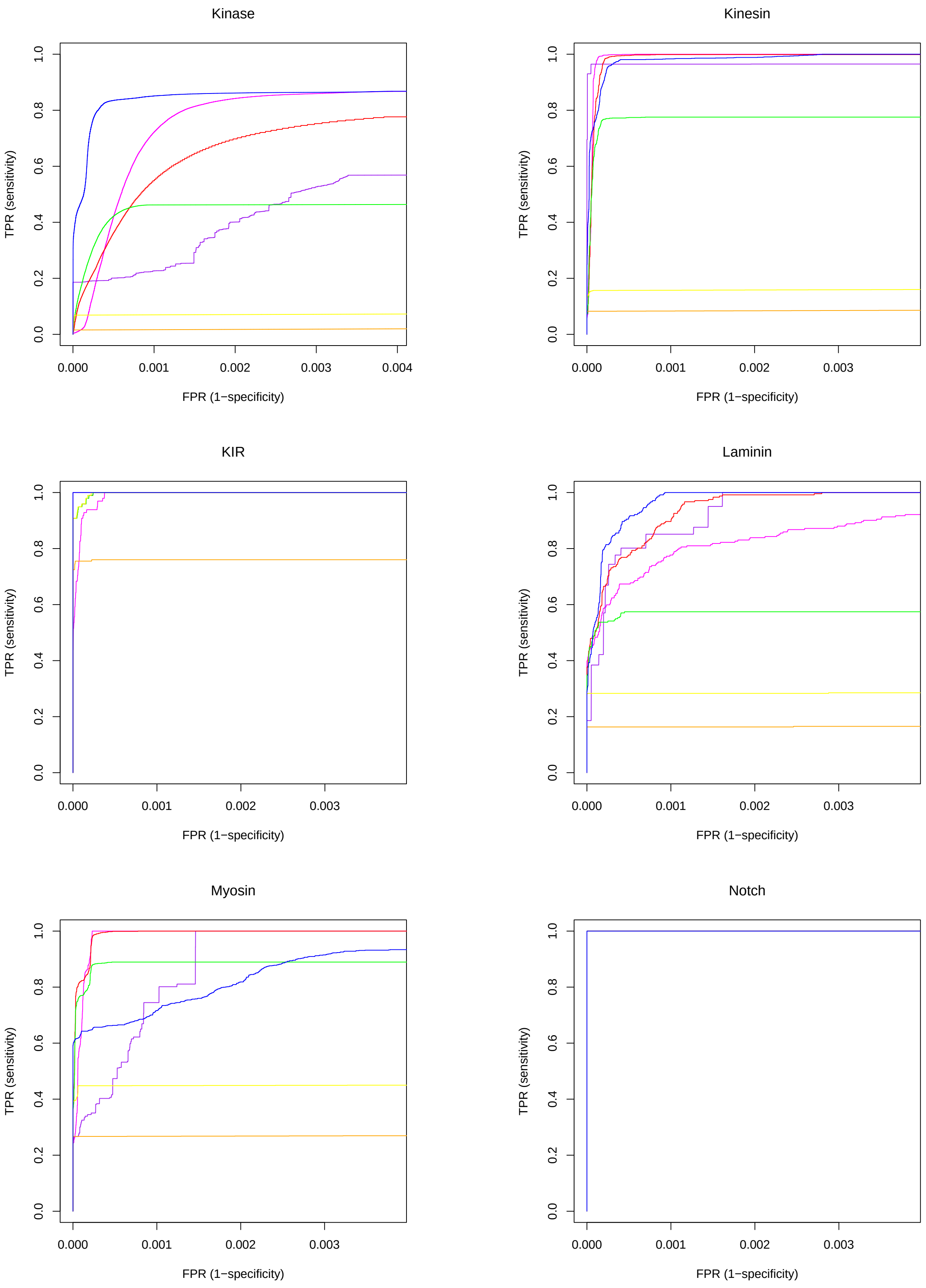
Kinase
Kinesin
KIR
Laminin
Myosin
Notch
TPR (sensitivity)
FPR (1−specificity)
0.0
0.2
0.4
0.6
0.8
1.0
0.000
0.001
0.002
0.003
0.004

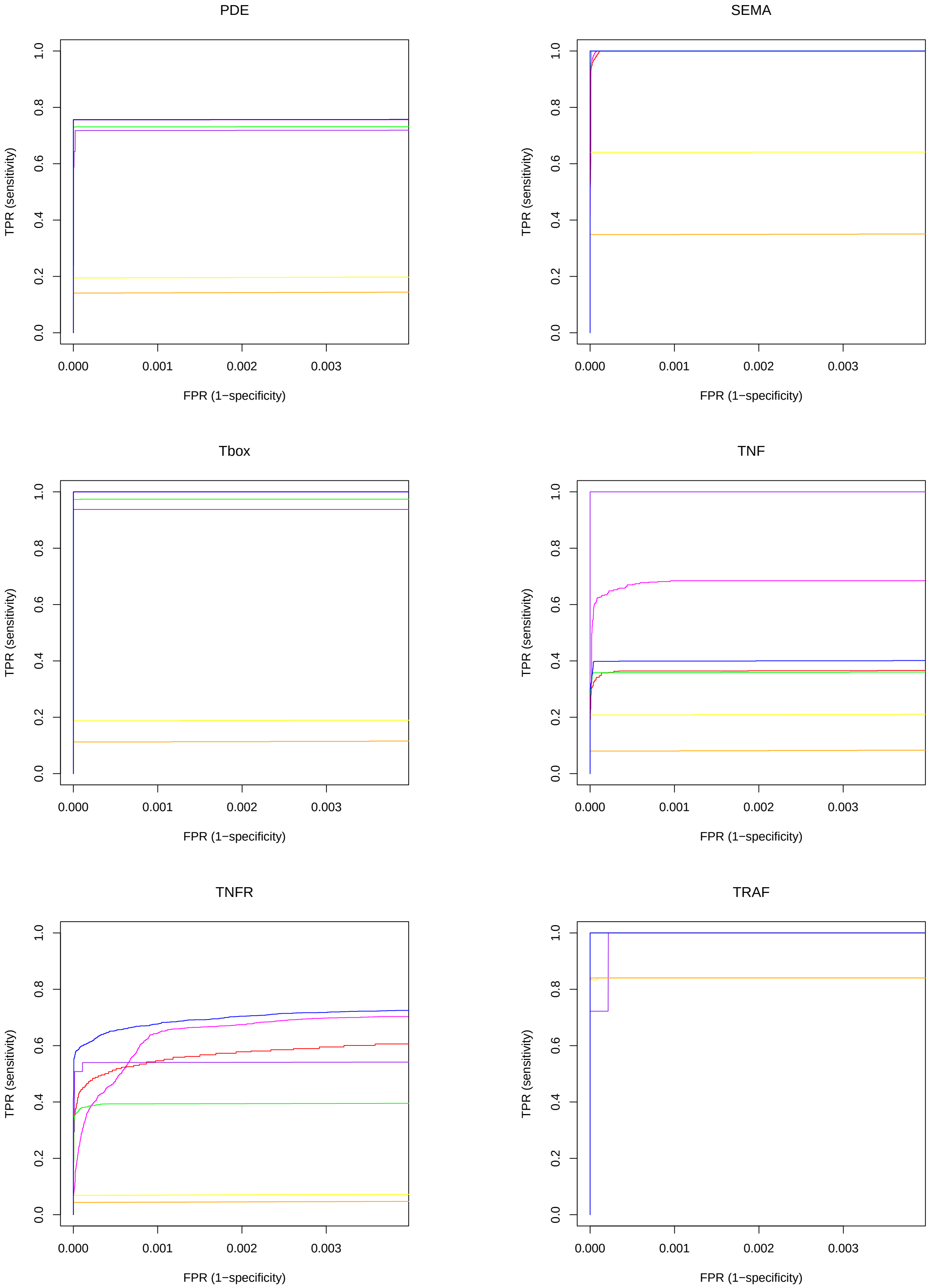
PDE
SEMA
Tbox
TNF
TNFR
TRAF
TPR (sensitivity)
FPR (1−specificity)
0.0
0.2
0.4
0.6
0.8
1.0
0.000
0.001
0.002
0.003

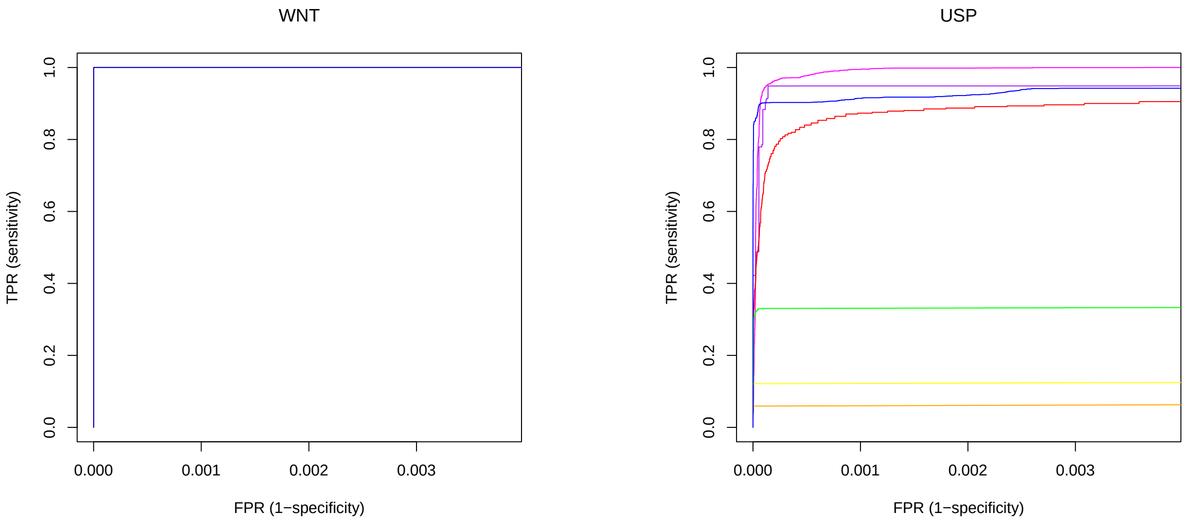

Figure S1: ROC-100k curves of Neighborhood Correlation (blue), BLAST (red), PSI-BLAST (magenta), DAC (purple) and alignment coverage ($\alpha \geq 0.3$: green, $\alpha \geq 0.6$: yellow, $\alpha \geq 0.8$: orange) for all families.
