# Supplementary material for: Sequence Similarity Network Reveals Common Ancestry of Multidomain Proteins
Source: PLoS Comput Biol. 2008 May 16;4(5):e1000063. doi: 10.1371/journal.pcbi.1000063 (PMC2377100; doi:10.1371/journal.pcbi.1000063)
Supplement: Figure S2 — Distributions of BLAST and NC scores for all families. (FF: blue, FO: red). (0.04 MB PDF) [file pcbi.1000063.s002.pdf]

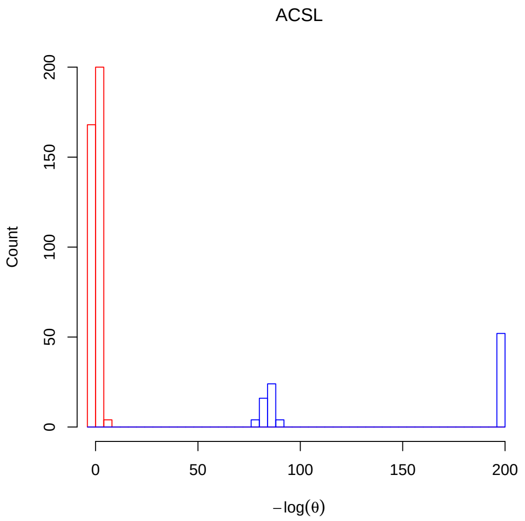
ACSL
Count
0
50
100
150
200
0
50
100
150
200
−log(θ)

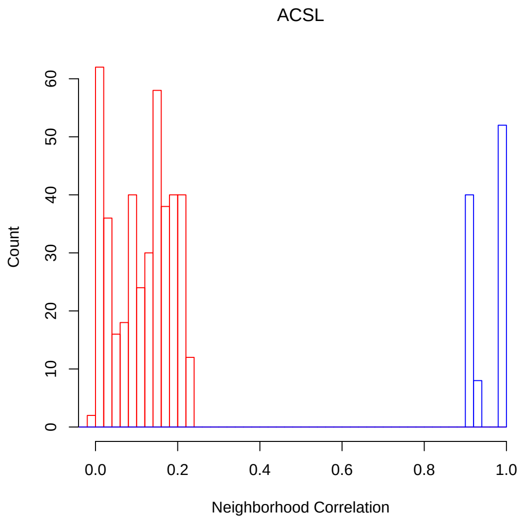
ACSL
Count
0
10
20
30
40
50
60
0.0
0.2
0.4
0.6
0.8
1.0
Neighborhood Correlation

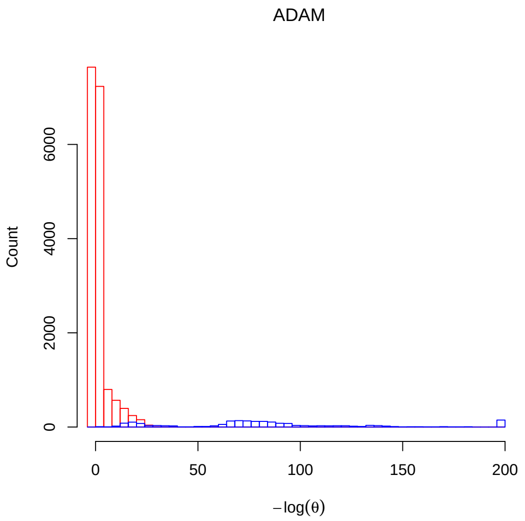
ADAM
Count
0
2000
4000
6000
0
50
100
150
200
−log(θ)

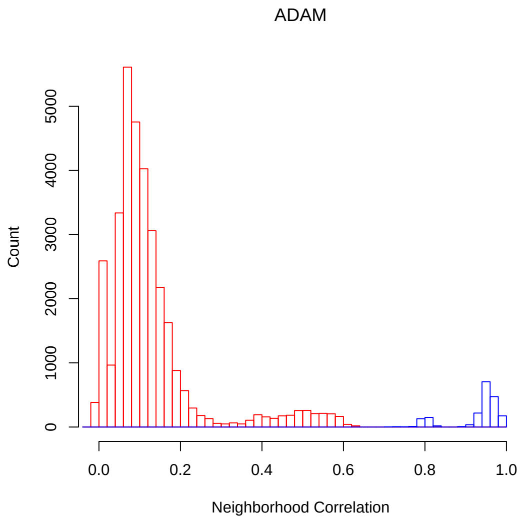
ADAM
Count
0
1000
2000
3000
4000
5000
0.0
0.2
0.4
0.6
0.8
1.0
Neighborhood Correlation

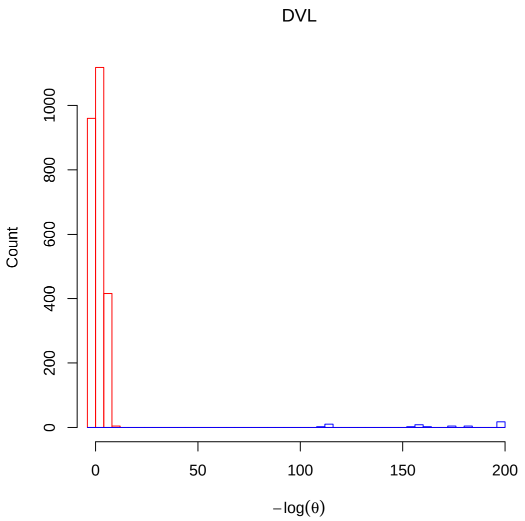
DVL
Count
0
200
400
600
800
1000
0
50
100
150
200
−log(θ)

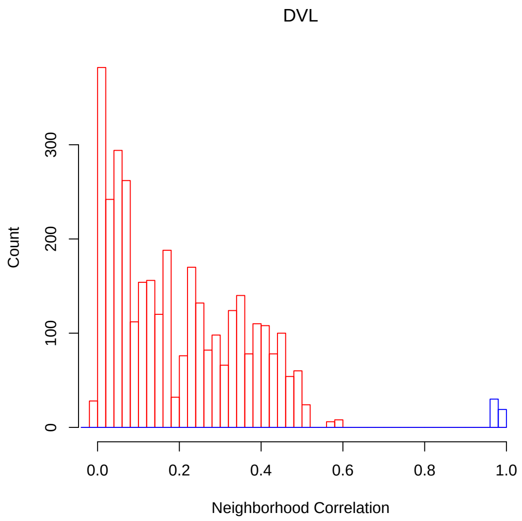
DVL
Count
0
100
200
300
0.0
0.2
0.4
0.6
0.8
1.0
Neighborhood Correlation

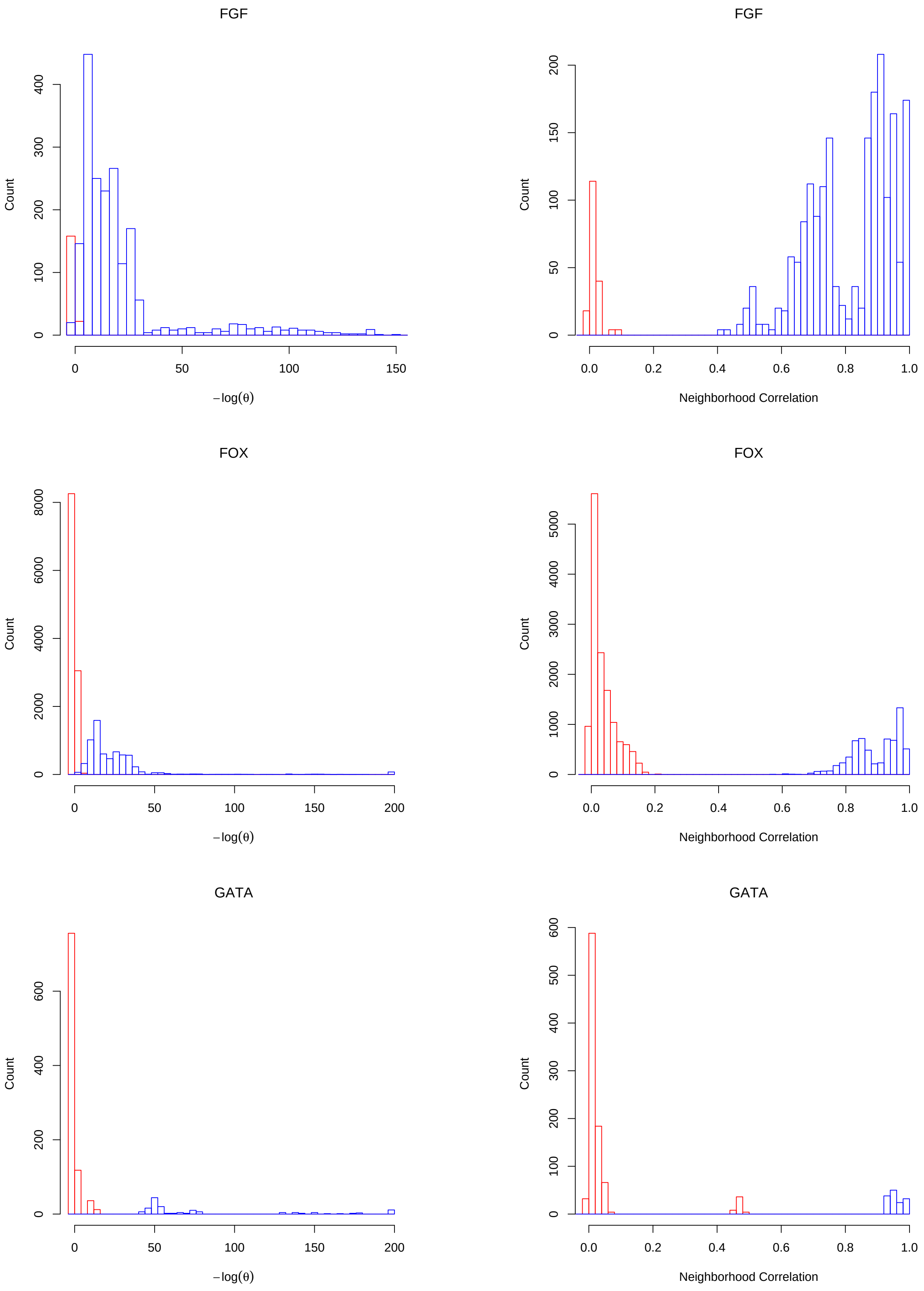
FGF
Count
0
100
200
300
400
0
50
100
150
−log(θ)
FGF
Count
0
50
100
150
200
0.0
0.2
0.4
0.6
0.8
1.0
Neighborhood Correlation
FOX
Count
0
2000
4000
6000
8000
0
50
100
150
200
−log(θ)
FOX
Count
0
1000
2000
3000
4000
5000
0.0
0.2
0.4
0.6
0.8
1.0
Neighborhood Correlation
GATA
Count
0
200
400
600
0
50
100
150
200
−log(θ)
GATA
Count
0
100
200
300
400
500
600
0.0
0.2
0.4
0.6
0.8
1.0
Neighborhood Correlation

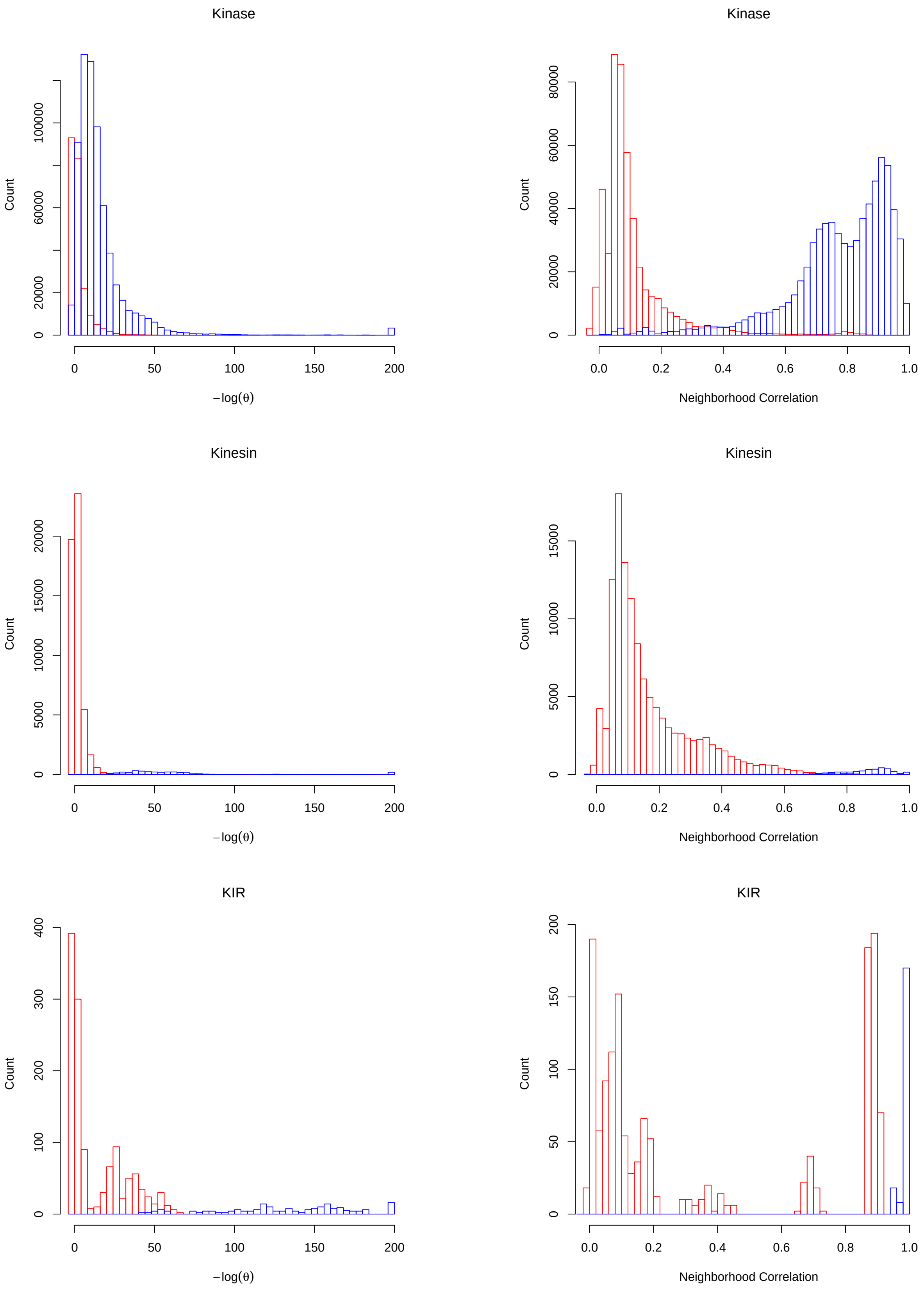
Kinase
Count
0
20000
60000
100000
0
50
100
150
200
−log(θ)
Kinase
Count
0
20000
40000
60000
80000
0.0
0.2
0.4
0.6
0.8
1.0
Neighborhood Correlation
Kinesin
Count
0
5000
10000
15000
20000
0
50
100
150
200
−log(θ)
Kinesin
Count
0
5000
10000
15000
0.0
0.2
0.4
0.6
0.8
1.0
Neighborhood Correlation
KIR
Count
0
100
200
300
400
0
50
100
150
200
−log(θ)
KIR
Count
0
50
100
150
200
0.0
0.2
0.4
0.6
0.8
1.0
Neighborhood Correlation

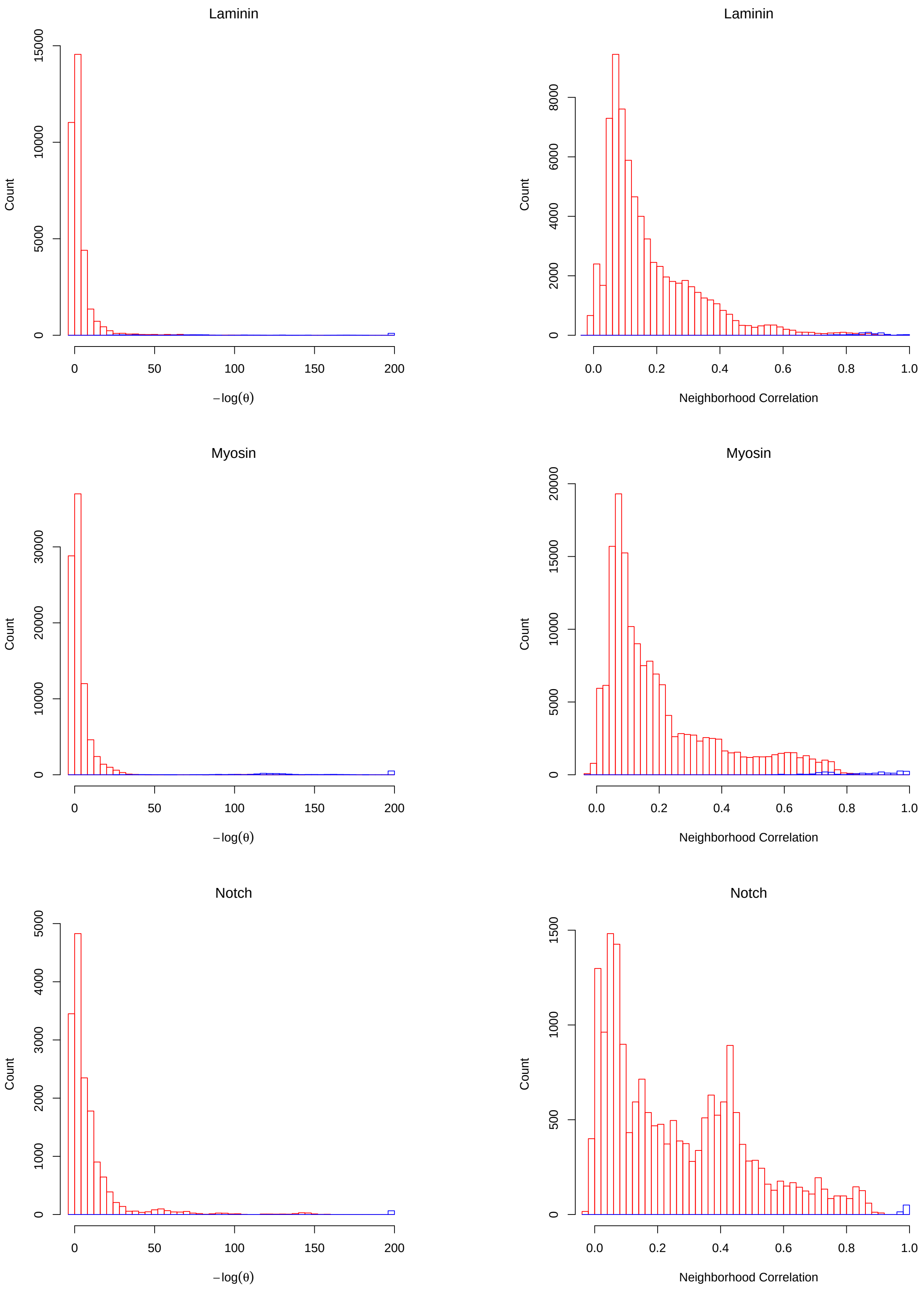
Laminin
Count
0
5000
10000
15000
0
50
100
150
200
−log(θ)
Laminin
Count
0
2000
4000
6000
8000
0.0
0.2
0.4
0.6
0.8
1.0
Neighborhood Correlation
Myosin
Count
0
10000
20000
30000
0
50
100
150
200
−log(θ)
Myosin
Count
0
5000
10000
15000
20000
0.0
0.2
0.4
0.6
0.8
1.0
Neighborhood Correlation
Notch
Count
0
1000
2000
3000
4000
5000
0
50
100
150
200
−log(θ)
Notch
Count
0
500
1000
1500
0.0
0.2
0.4
0.6
0.8
1.0
Neighborhood Correlation

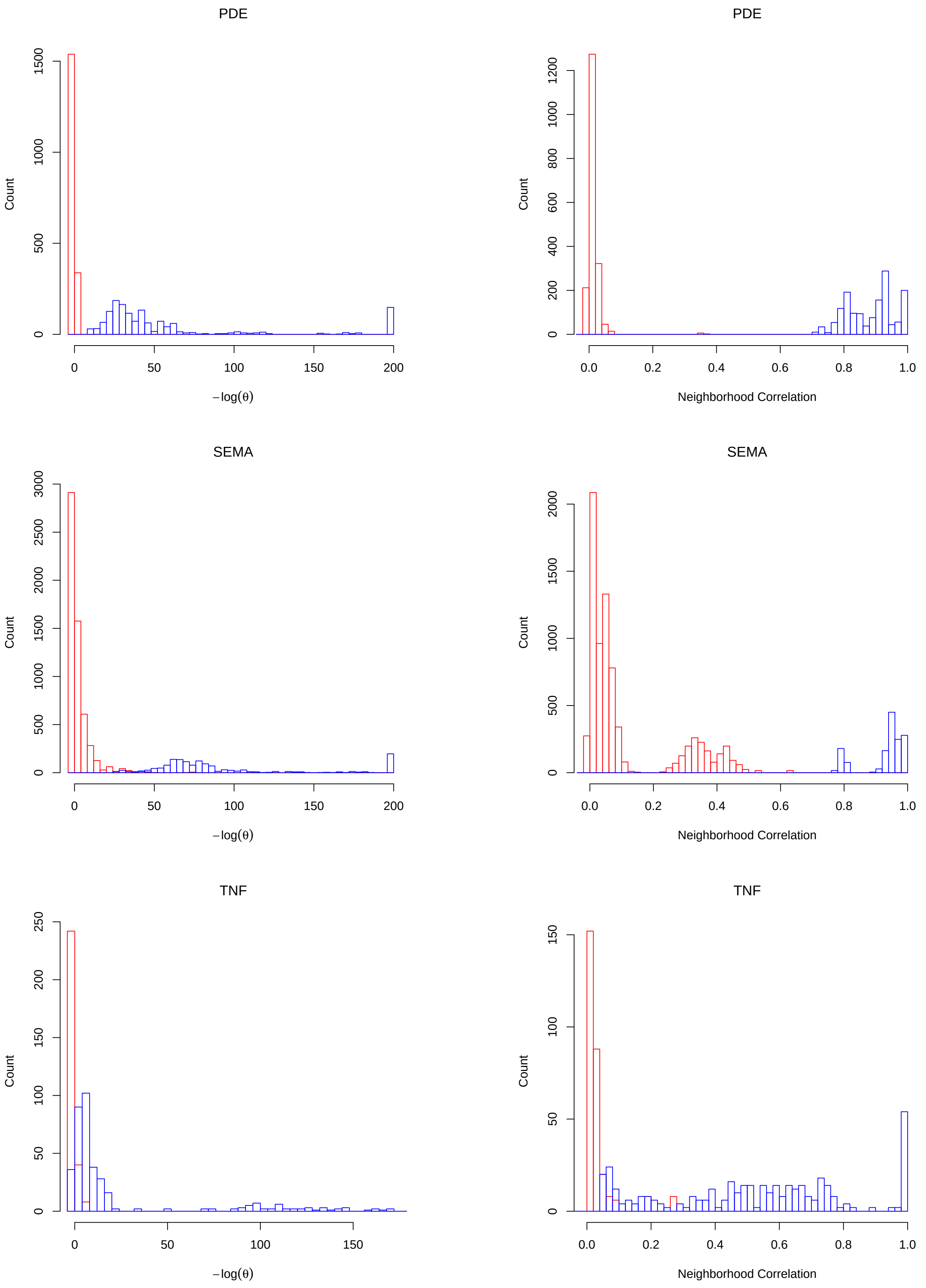
PDE
Count
$-\log(\theta)$
PDE
Count
Neighborhood Correlation
SEMA
Count
$-\log(\theta)$
SEMA
Count
Neighborhood Correlation
TNF
Count
$-\log(\theta)$
TNF
Count
Neighborhood Correlation

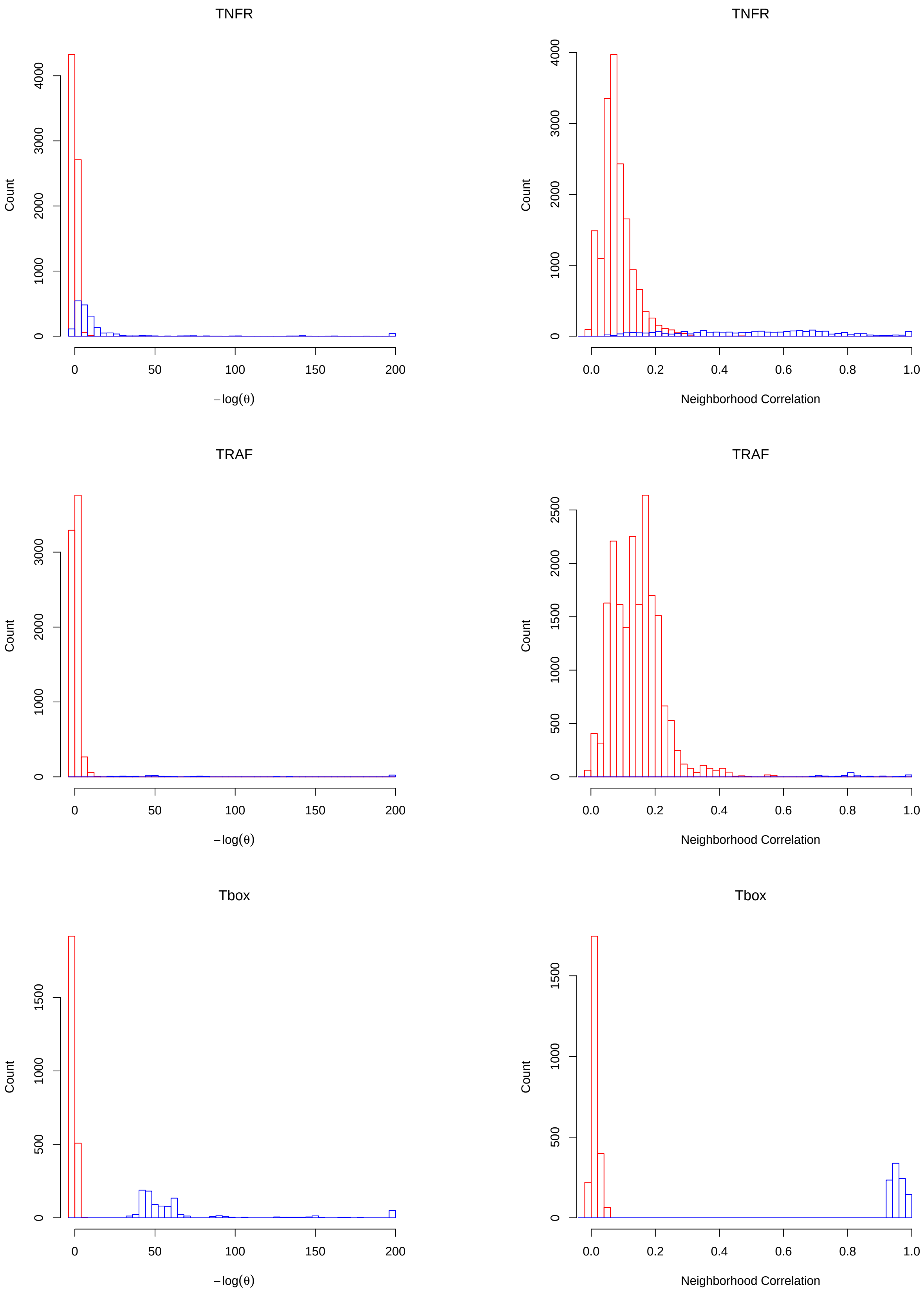
TNFR
Count
0
1000
2000
3000
4000
0
50
100
150
200
−log(θ)
TNFR
Count
0
1000
2000
3000
4000
0.0
0.2
0.4
0.6
0.8
1.0
Neighborhood Correlation
TRAF
Count
0
1000
2000
3000
0
50
100
150
200
−log(θ)
TRAF
Count
0
500
1000
1500
2000
2500
0.0
0.2
0.4
0.6
0.8
1.0
Neighborhood Correlation
Tbox
Count
0
500
1000
1500
0
50
100
150
200
−log(θ)
Tbox
Count
0
500
1000
1500
0.0
0.2
0.4
0.6
0.8
1.0
Neighborhood Correlation

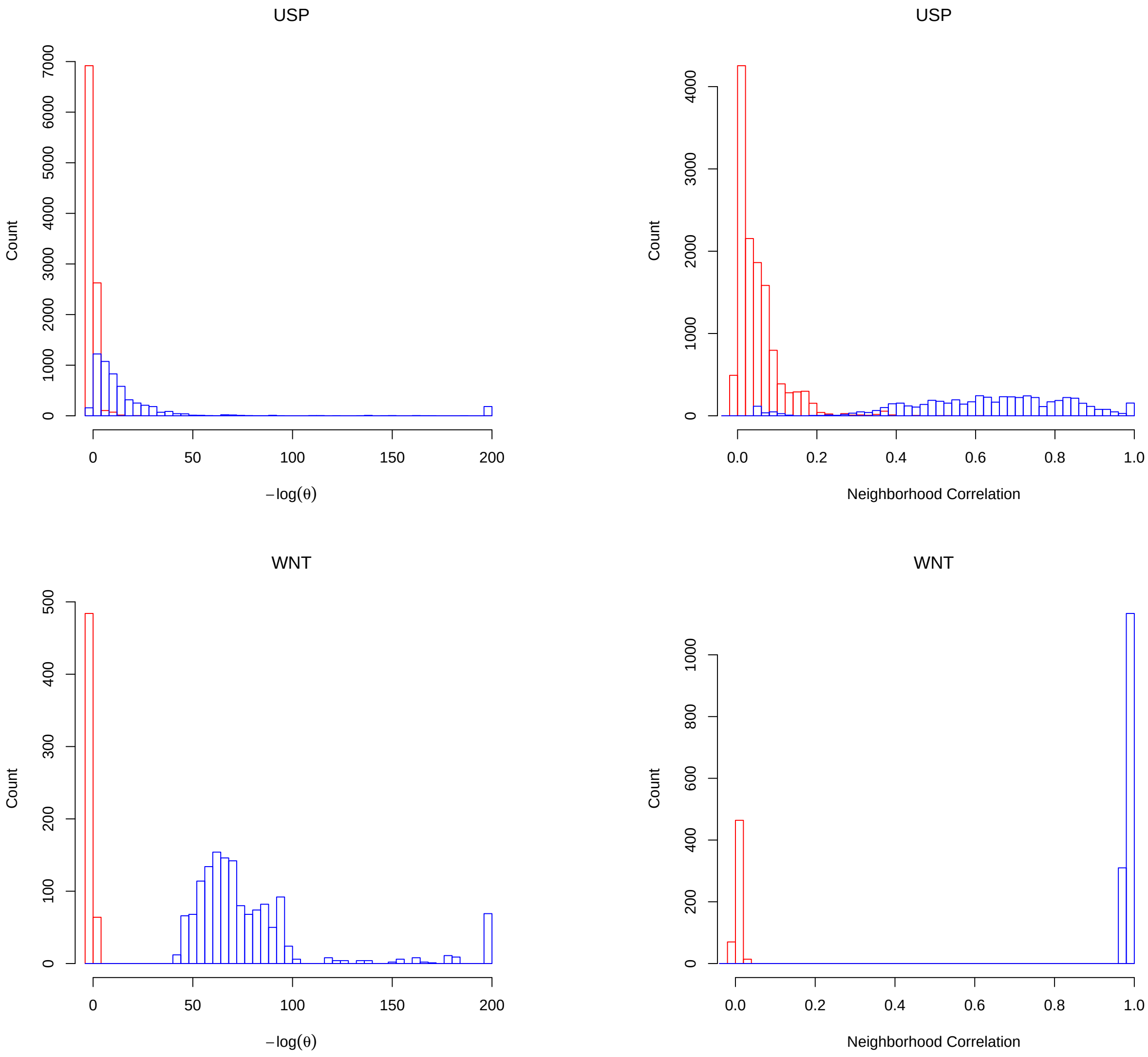

Figure S2: Distributions of significance and NC scores for all families. FF matches are shown in blue, FO matches in red.
