# Supplementary figures and images for: Sequence Similarity Network Reveals Common Ancestry of Multidomain Proteins
Source: PLoS Comput Biol. 2008 May 16;4(5):e1000063. doi: 10.1371/journal.pcbi.1000063 (PMC2377100; doi:10.1371/journal.pcbi.1000063)

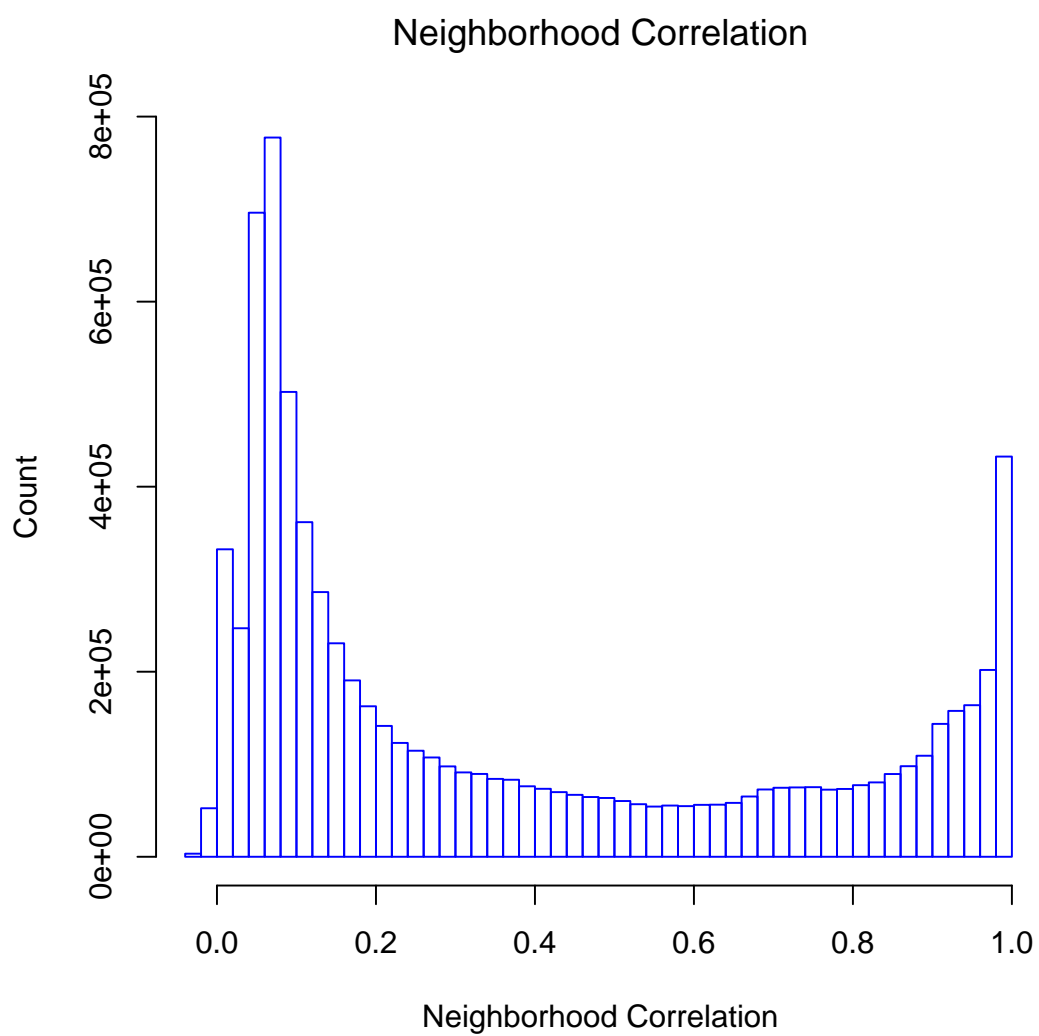

Figure S3: Distribution of NC scores for all sequence pairs.

Supplement: Figure S3 — Distribution of Neighborhood Correlation scores for all sequence pairs. (0.00 MB PDF) [file pcbi.1000063.s003.pdf]
