# Supplementary material for: Sequence Similarity Network Reveals Common Ancestry of Multidomain Proteins
Source: PLoS Comput Biol. 2008 May 16;4(5):e1000063. doi: 10.1371/journal.pcbi.1000063 (PMC2377100; doi:10.1371/journal.pcbi.1000063)
Supplement: Figure S4 — Distributions of alignment coverage for all families. Distributions of alignment coverage calculated with the optimal alignment length only (FF: blue, FO: red) and with combined non-conflicting alignments (FF: turquoise, FO: brown) for all families. (0.03 MB PDF) [file pcbi.1000063.s004.pdf]

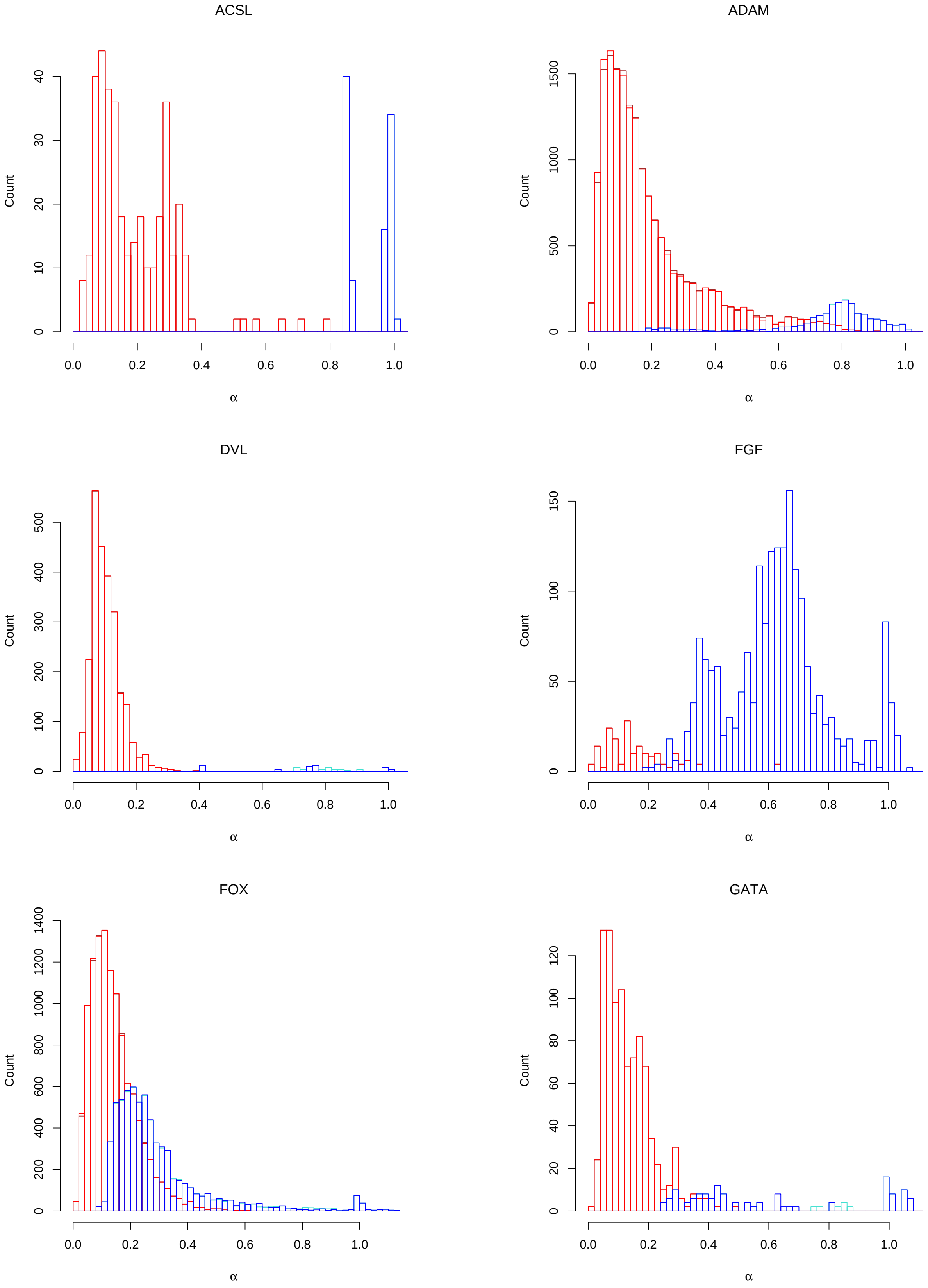
ACSL
ADAM
DVL
FGF
FOX
GATA
Count
α
0.0
0.2
0.4
0.6
0.8
1.0
0
10
20
30
40
500
1000
1500
100
200
300
400
50
150
600
800
1200
1400
60
80
120

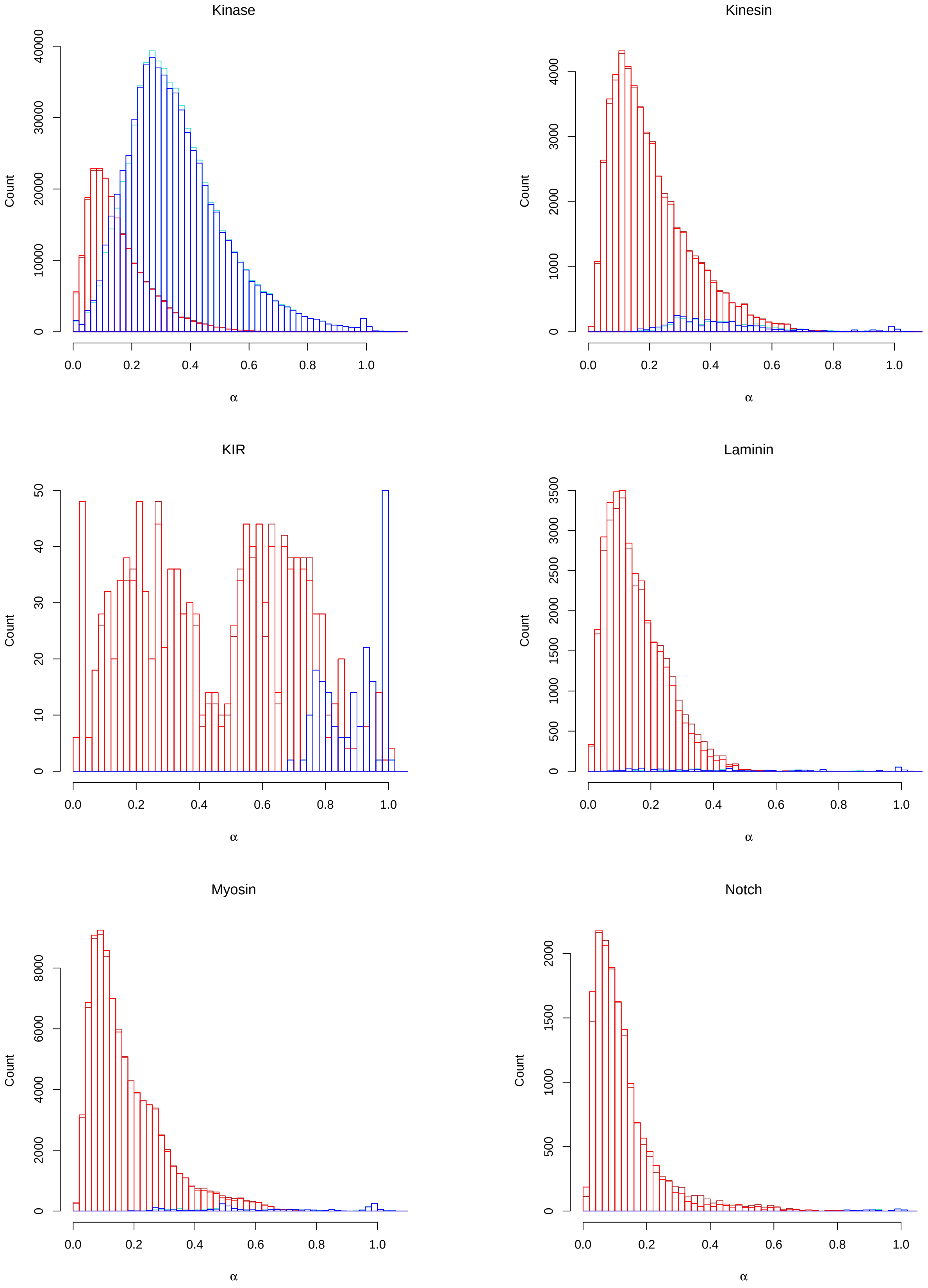
Kinase
Count
0
10000
20000
30000
40000
0.0
0.2
0.4
0.6
0.8
1.0
α
Kinesin
Count
0
1000
2000
3000
4000
0.0
0.2
0.4
0.6
0.8
1.0
α
KIR
Count
0
10
20
30
40
50
0.0
0.2
0.4
0.6
0.8
1.0
α
Laminin
Count
0
500
1000
1500
2000
2500
3000
3500
0.0
0.2
0.4
0.6
0.8
1.0
α
Myosin
Count
0
2000
4000
6000
8000
0.0
0.2
0.4
0.6
0.8
1.0
α
Notch
Count
0
500
1000
1500
2000
0.0
0.2
0.4
0.6
0.8
1.0
α

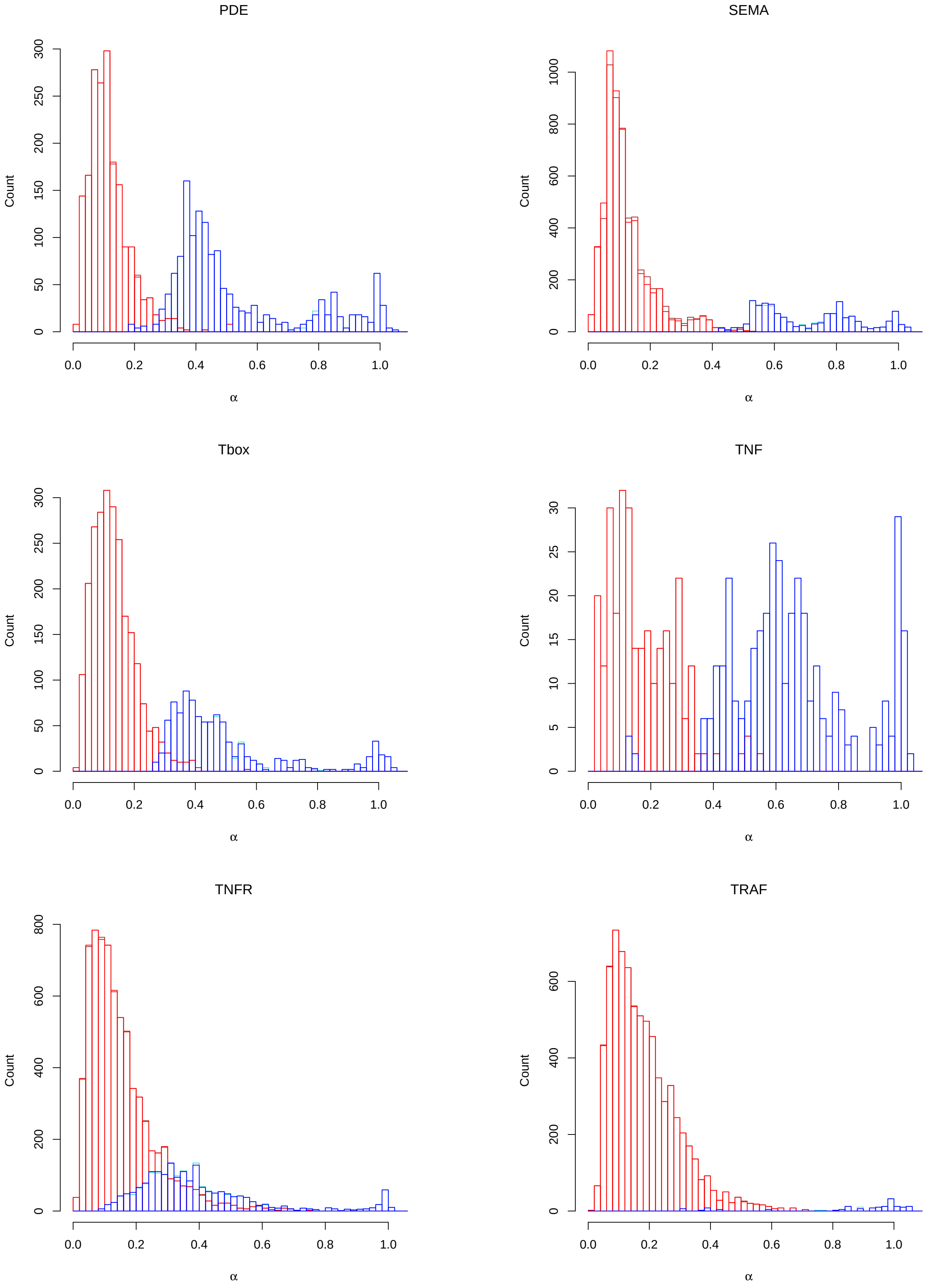
PDE
SEMA
Tbox
TNF
TNFR
TRAF
Count
α
0.0
0.2
0.4
0.6
0.8
1.0

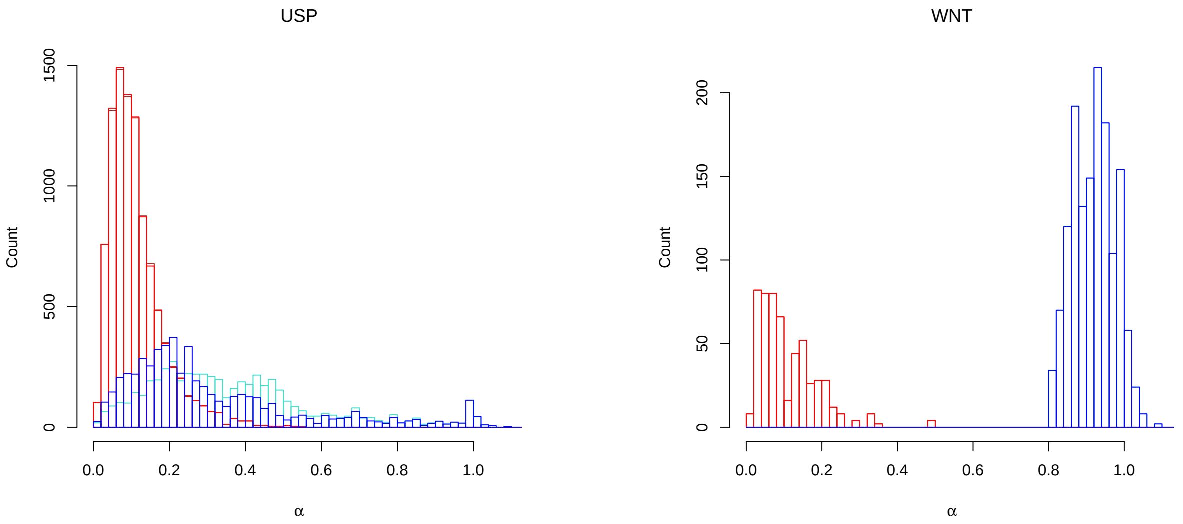

Figure S4: Distributions of alignment coverage calculated with the optimal alignment length only (FF: blue, FO: red) and with combined non-conflicting alignments (FF: turquoise, FO: brown) for all families.
