# Supplementary material for: Sequence Similarity Network Reveals Common Ancestry of Multidomain Proteins
Source: PLoS Comput Biol. 2008 May 16;4(5):e1000063. doi: 10.1371/journal.pcbi.1000063 (PMC2377100; doi:10.1371/journal.pcbi.1000063)
Supplement: Table S1 — Precision and Recall for predictions using simple alignment coverage thresholds of 0.3, 0.6, and 0.8 for all families. (0.07 MB DOC) [file pcbi.1000063.s005.doc]

|  | $\alpha\geq0.3$ | | $\alpha\geq0.6$ | | $\alpha\geq0.8$ | |
| --- | --- | --- | --- | --- | --- | --- |
|  | Precision | Recall | Precision | Recall | Precision | Recall |
| *ALL* | 0.8810 | 0.4675 | 0.9556 | 0.0772 | 0.9893 | 0.0217 |
| *ALL-Kin* | 0.3775 | 0.6072 | 0.7853 | 0.2904 | 0.9758 | 0.1732 |
| Single Domain Families | | | | | | |
| ACSL | 0.6329 | 1.0000 | 0.9434 | 1.0000 | 1.0000 | 1.0000 |
| FGF | 0.9906 | 0.9752 | 0.9966 | 0.5992 | 1.0000 | 0.1384 |
| FOX | 0.7939 | 0.3160 | 0.9951 | 0.0616 | 1.0000 | 0.0291 |
| Tbox | 0.9303 | 0.9729 | 1.0000 | 0.1863 | 1.0000 | 0.1113 |
| TNF | 0.9196 | 0.3574 | 1.0000 | 0.2070 | 1.0000 | 0.0791 |
| USP | 0.8828 | 0.3304 | 1.0000 | 0.1213 | 1.0000 | 0.059 |
| WNT | 0.9904 | 1.0000 | 1.0000 | 1.0000 | 1.0000 | 1.0000 |
| Mean | 0.8772 | 0.7074 | 0.9907 | 0.4536 | 1.0000 | 0.3453 |
| Multidomain Families, Conserved Architecture | | | | | | |
| DVL | 0.8596 | 1.0000 | 1.0000 | 0.7551 | 1.0000 | 0.2449 |
| GATA | 0.7949 | 0.8611 | 1.0000 | 0.4028 | 1.0000 | 0.3056 |
| Notch | 0.0769 | 1.0000 | 0.4571 | 1.0000 | 0.9697 | 1.0000 |
| KIR | 0.1948 | 1.0000 | 0.3256 | 1.0000 | 0.6435 | 0.7551 |
| TRAF | 0.1265 | 1.0000 | 0.8108 | 0.8333 | 0.9836 | 0.8333 |
| Mean | 0.4105 | 0.9722 | 0.7187 | 0.7982 | 0.9194 | 0.6278 |
| Multidomain Families, Variable Architecture | | | | | | |
| ADAM | 0.3666 | 0.9452 | 0.7265 | 0.8781 | 0.9602 | 0.4731 |
| Kinase | 0.9470 | 0.4619 | 0.9917 | 0.0687 | 0.9601 | 0.0157 |
| Kinesin | 0.1886 | 0.7755 | 0.4731 | 0.1569 | 0.9416 | 0.0823 |
| Laminin | 0.1055 | 0.5744 | 0.9714 | 0.281 | 1.0000 | 0.1612 |
| Myosin | 0.1340 | 0.8894 | 0.5284 | 0.448 | 1.0000 | 0.2665 |
| PDE | 0.9698 | 0.7304 | 1.0000 | 0.1942 | 1.0000 | 0.1405 |
| SEMA | 0.8347 | 1.0000 | 1.0000 | 0.6399 | 1.0000 | 0.3476 |
| TNFR | 0.6669 | 0.3931 | 0.8734 | 0.0684 | 1.0000 | 0.0433 |
| Mean | 0.5266 | 0.7212 | 0.8205 | 0.3419 | 0.9871 | 0.1913 |

Table S1: Precision and Recall for predictions using alignment coverage thresholds of

0.3, 0.6, and 0.8 for all families.
