# Supplementary material for: Sequence Similarity Network Reveals Common Ancestry of Multidomain Proteins
Source: PLoS Comput Biol. 2008 May 16;4(5):e1000063. doi: 10.1371/journal.pcbi.1000063 (PMC2377100; doi:10.1371/journal.pcbi.1000063)
Supplement: Table S2 — Precision and recall for predictions using combined alignment coverage thresholds of 0.3, 0.6, and 0.8 for all families. (0.07 MB DOC) [file pcbi.1000063.s006.doc]

|  | $\alpha\geq0.3$ | | $\alpha\geq0.6$ | | $\alpha\geq0.8$ | |
| --- | --- | --- | --- | --- | --- | --- |
|  | Precision | Recall | Precision | Recall | Precision | Recall |
| *ALL* | 0.8776 | 0.4777 | 0.9549 | 0.0787 | 0.9889 | 0.0220 |
| *ALL-Kin* | 0.3807 | 0.6528 | 0.7861 | 0.2999 | 0.9750 | 0.1771 |
| Single Domain Families | | | | | | |
| ACSL | 0.6329 | 1.0000 | 0.9434 | 1.0000 | 1.0000 | 1.0000 |
| FGF | 0.9906 | 0.9752 | 0.9966 | 0.5992 | 1.0000 | 0.1384 |
| FOX | 0.7943 | 0.3190 | 0.9954 | 0.0663 | 1.0000 | 0.0352 |
| Tbox | 0.9303 | 0.9729 | 1.0000 | 0.1883 | 1.0000 | 0.1124 |
| TNF | 0.9196 | 0.3574 | 1.0000 | 0.2070 | 1.0000 | 0.0791 |
| USP | 0.9188 | 0.5001 | 1.0000 | 0.1375 | 1.0000 | 0.0612 |
| WNT | 0.9904 | 1.0000 | 1.0000 | 1.0000 | 1.0000 | 1.0000 |
| Mean | 0.8824 | 0.7321 | 0.9908 | 0.4569 | 1.0000 | 0.3466 |
| Multidomain Families, Conserved Architecture | | | | | | |
| DVL | 0.8596 | 1.0000 | 1.0000 | 1.0000 | 1.0000 | 0.6735 |
| GATA | 0.7949 | 0.8611 | 1.0000 | 0.4028 | 1.0000 | 0.3611 |
| Notch | 0.0499 | 1.0000 | 0.3951 | 1.0000 | 0.9697 | 1.0000 |
| KIR | 0.1948 | 1.0000 | 0.3203 | 1.0000 | 0.6325 | 0.7551 |
| TRAF | 0.1263 | 1.0000 | 0.8205 | 0.8889 | 0.9841 | 0.8611 |
| Mean | 0.4051 | 0.9722 | 0.7072 | 0.8583 | 0.9173 | 0.7302 |
| Multidomain Families, Variable Architecture | | | | | | |
| ADAM | 0.3635 | 0.9452 | 0.7234 | 0.8781 | 0.9603 | 0.4742 |
| Kinase | 0.9456 | 0.4707 | 0.9912 | 0.0699 | 0.9953 | 0.0158 |
| Kinesin | 0.1973 | 0.8355 | 0.5095 | 0.1875 | 0.9296 | 0.0842 |
| Laminin | 0.1166 | 0.8306 | 0.9756 | 0.3306 | 1.0000 | 0.1983 |
| Myosin | 0.1368 | 0.9537 | 0.5306 | 0.4584 | 1.0000 | 0.2732 |
| PDE | 0.9698 | 0.7304 | 1.0000 | 0.1942 | 1.0000 | 0.1405 |
| SEMA | 0.8242 | 1.0000 | 1.0000 | 0.6510 | 1.0000 | 0.3476 |
| TNFR | 0.6691 | 0.3983 | 0.8745 | 0.0691 | 1.0000 | 0.0433 |
| Mean | 0.5279 | 0.7705 | 0.8256 | 0.3549 | 0.9857 | 0.1971 |

Table S2: Precision and Recall for predictions using combined alignment coverage thresholds of 0.3, 0.6, and 0.8 for all families.
