# Supplementary material for: Sequence Similarity Network Reveals Common Ancestry of Multidomain Proteins
Source: PLoS Comput Biol. 2008 May 16;4(5):e1000063. doi: 10.1371/journal.pcbi.1000063 (PMC2377100; doi:10.1371/journal.pcbi.1000063)
Supplement: Text S1 — Supporting Text. (0.09 MB DOC) [file pcbi.1000063.s008.doc]

## **Supporting Information**

### Suboptimal alignment heuristic

To take suboptimal alignments into account, we used a simple heuristic method for selecting a set of high-scoring local alignments that do not conflict. Two alignments conflict if they overlap or do not appear in the same order in both sequences. More formally, let $a^{i}(x,y)=\{(x_{s}^{i},x_{e}^{i}),(y_{s}^{i},y_{e}^{i})\}$ be the *i^th^* local alignment, where $x_{s}^{i}$ and $x_{e}^{i}$ are the starting and ending positions of the alignment in sequence *x.* Similarly $y_{s}^{i}$ and $y_{e}^{i}$ are the starting and ending positions of the alignment in *y.* Two alignments a*^i^* and a*^j^* do not conflict if $x_{s}^{i}>x_{e}^{i}$ and $y_{s}^{i}>y_{e}^{i}$ or $x_{s}^{i}<x_{e}^{i}$ and$y_{s}^{i}<y_{e}^{i}$. The heuristic for finding a set of non-conflicting, local alignments between sequences *x* and *y* proceeds as follows:

Let $\varsigma$= Ф
Let *C* = {*a^i^*(*x*, *y*)|*S*(*a^i^*(*x*, *y*)) > threshold}
While (*C* = Ф){

Let a*^i^*(*x*, *y*) be an alignment in *C* with maximum score.
Remove a*^i^*(*x*, *y*) from *C* and place it in *S* .
Remove all alignments from *C* that conflict a*^i^*(*x*, *y*).

}

The length of the region of similarity is then calculated by summing the lengths of the alignments in$\varsigma$.

Curation of test families

We curated a sequence benchmark for evaluation of homology detection methods, with an emphasis on multidomain homology. This test set is constructed from amino acid sequences drawn from twenty well-studied sequence families. For each family, we derived a list of designated gene symbols, Pfam [1] and/or InterPro [2] codes from publications by family experts, and reports from standards committees, such as the HUGO Gene Nomenclature Committee[[1]](#footnote-2). When the symbols of a family have been standardized, symbols are good criteria for family identification. For protein families that are characterized by specific domains, domain information can also used to identify protein family members.

These lists were used to generate a preliminary roster for each family. We further confirmed family membership by referring to the evolution of specific gene families from the literature. The identification criteria and references used to confirm each family are given below. The complete set of SwissProt [3] accessions for each family is given in the next section.

**Acyl-CoA synthetase long-chain (ACSL):** Long chain acyl-CoA synthetases are members of an ancient superfamily of AMP-binding proteins involved in lipid metabolism [4]. Unlike other acyl-CoA synthetases, ACSLs primarily catalyze long-chain fatty acids. Eukaryotic ACSLs are uniquely characterized by a 30 to 70 residue linker sequence that is not present in bacterial ACSLs or in AMP-binding proteins that catalyze short-, medium- or very long-chain fatty acids [5]. The presence of this linker region, combined with a conserved single-domain architecture, supports common ancestry for this family.
**Initial screen:** Symbol list derived from HGNC.
**References:** [4, 6]

**A Disintegrin and Metalloprotease (ADAM):** ADAMs are zinc-dependent metalloproteases thought to carry out functions such as cell adhesion, fusion, signaling, and proteolysis in the context of processes including fertilization, development, and inflammation [7]. All ADAM sequences are characterized by a conserved extracellular domain architecture that includes metalloprotease, disintegrin, and cysteine-rich domains. Many ADAM sequences also contain EGF-like domains. Both the cysteine-rich and EGF-like domains are promiscuous and cause ADAMs to match unrelated sequences such as Tenascin, Notch, Integrin, and some Kinases. ADAMs have not been observed in yeast or bacteria, but do occur in fly and worm and, in much larger numbers, in human. The N-terminal domain architecture conserved in all ADAM proteins supports common ancestry for the family.
**Initial screen:** Symbol lists derived from HGNC.
**References:** [8­-12]

**Dishevelled (DVL):** DVL is a small family of multidomain proteins that participate in the WNT pathway, a developmental pathway that mediates cell-cell interactions during embryogenesis [13]. DVL domain architecture is largely conserved. In our dataset, all members of this family have DEP, DIX, and PDZ domains. DVL proteins
are adapters with many binding partners. This variety of interactions is achieved through its multidomain architecture, in which each domain is responsible for a specific set of interactions. Both the PDZ and the DEP domain occur in a number of unrelated signaling proteins. For this reason, DVL proteins match a large number
of sequences outside the family.

DVL homologs have been found in fly, worm, mouse, and human, but not in yeast or bacteria, suggesting that this family arose in metazoans. Both DVL sequence and domain architecture are highly conserved. This conservation, along with the small size of the family, suggest that the family arose through duplication of a single progenitor sequence.
**Initial screen:** Symbol list derived from HGNC.
**References:** [13, 14]

**Fibroblast Growth Factor (FGF):** FGF is a single domain family of developmental proteins involved in regulating cell differentiation, proliferation, and migration during embryogenesis as well as in tissue repair in adults [15]. In human, there are seven FGF subfamilies, each with similar biochemical and developmental properties. This
family has a conserved core roughly 120 amino acids in length with 30% to 60% identity within the core [15]. Overall, FGF sequences have low conservation, although sequence similarity is greater within each subfamily. FGF sequences have been only been observed in metazoans [16]. Phylogenetic analysis, exon-intron structure, and conserved synteny support the hypothesis that the vertebrate FGF gene family expanded by gene duplication in two phases, in early metazoan and early vertebrate evolution, respectively [16].
**Initial screen:** FGF proteins are uniquely identified by gene symbols with the prefix FGF [15].
**References:** [15­-17]

**Forkhead box (FOX):** FOX is a family of transcription factors involved in the regulation of developmental processes, including tissue determination, differentiation, and homeostasis [18,19]. FOX genes have been observed in yeast and animals but not in plants. FOX proteins share a conserved winged helix DNA-binding domain approximately 110 residues in length. Family members differ in their transactivation and transrepression domains; regions outside the conserved domain are highly divergent. Most FOX proteins have single-domain architectures, but a few also contain the promiscuous domain, FHA.

**Initial screen:** FOX proteins are uniquely identified by gene symbols with the prefix FOX [19] and HGNC.
**References:** [19-­21]

**GATA binding protein (GATA):** GATA is a family of transcription factors that play a role in determining cell fates during development. All six vertebrate GATA proteins have two tandem zinc finger domains. In addition, GATA 4/5/6 have an N-terminal transactivation domain that is unique to chordates [22, 23]. According to
phylogenetic evidence, these six genes were generated by duplication from a common ancestral gene [23].
**Initial screen:** GATA proteins are uniquely identified by gene symbols with the prefix GATA [23].
**References:** [22, 23]

**Kinases:** The Kinases [24, 25], one of the largest known families, are involved in many aspects of cellular control, including cell cycle progression, signal transduction, metabolism, and cell movement. Kinases achieve this variety of cellular activity through modular domain architectures. All Kinases share a kinase domain derived from a common ancestor [26, 27]. In addition to the kinase domain, which mediates phosphorylation, many Kinases have additional domains for recognition of binding partners. The repertoire of such domains is large and diverse and many appear in other protein families as well. In our dataset, more than 100 non-kinase domains are found in combination with kinase domains, according to the CDD database [28].

Several lines of evidence support common ancestry for the Kinase family. All kinase domains share a common origin [26, 27]. In addition, there is evidence to suggest that kinase domains are not mobile; that is that, multidomain Kinases arose through insertion of other domains into existing Kinase genes. Tordai *et al.* have observed that mobile domains tend to be small and have 1-1 intron phase [29]. The pkinase domain is larger than typical promiscuous domains and is not 1-1. Moreover, mobile domains typically have auxiliary functions such as adapter and adhesion domains [29]. In contrast, pkinase has enzymatic function. Finally, roughly 40% of Kinases are single domain proteins [30]. These occur primarily in more ancient Kinase subfamilies (AGC, CAMK, CK1, STE, and CMGC). Single domain Kinases are relatively rare in the more recent TK and TKL subfamilies, which are specific to metazoans. This is consistent with the hypothesis that single domain Kinases represent the ancestral state and that multidomain Kinases arose through insertion of mobile domains into existing Kinases.
**Initial screen:** Symbol list derived from [24, 31]. The Pfam codes in PF07714, PF00069, PF06293, PF03881, PF02958, PF07914, PF01633, PF04655, PF01636, PF03109, PF05445, PF01163, and PF06176 are Kinase-specific [26]. If a sequence had either a symbol or a Pfam code from our candidate lists, it was placed on the preliminary Kinase roster.
**References:** [24­-26, 30­-41]

**Kinesins:** A family of microtubule-associated molecular motors that are transport regulators for organelles and vesicles, Kinesins are involved in cell division and play a role in pattern formation in embryogenesis. All Kinesins share a conserved motor domain ranging in length from 340 to 450 residues [42, 43]. A few family members contain other domains, including a number of coiled-coil (e.g., MAD, SF-assemblin, HOOK, Myosin-tail-1) and/or promiscuous domains (e.g., FHA, PH). It has been proposed that Kinesins evolved through a first round of duplication of a simple Kinesin, followed by domain insertions which formed the progenitors of the 14
modern Kinesin subfamilies. Additional duplications resulted in proliferation within these subfamilies [42, 44, 45].
**Initial screen:** Symbol list derived from [46] and HGNC. If a sequence had both a symbol in our candidate list and the Kinesin-specific InterPro code, IPR001752, it was placed on the preliminary Kinesin roster.
**References:** [42, 46­-49]

**Killer cell Immunoglobulin-like Receptors (KIR):** A family of type-1 transmembrane glycoproteins active in innate immune response through regulation of natural killer cells and some T cells, KIR proteins have an extra-cellular ligand-binding region containing two or three Ig domains and an intracellular portion responsible for signal inhibition or activation [50]. This family is largely specific to primates: Only two KIR-like genes have been identified in mouse and it is not known whether these are functional [51]. The analogous function in mouse is carried out by the lectin-resembling Ly49 receptors [52].

The pattern of gene family evolution observed in the KIR family is unique within our test set [53, 54]. The KIR family appears to have descended from an ancestral gene in the primate/rodent ancestor. Many KIR genes are specific to a small number of primate species, suggesting lineage-specific duplication and specialization.
Human KIR genes are arranged in a tandem cluster on chromosome 19. Repetitive sequences are dispersed throughout this region, in both intergenic and intronic DNA, conferring an extraordinary degree of plasticity on the region. The number of KIR genes varies within the human population, consistent with frequent changes in
copy number due to unequal crossing over. Moreover, there is evidence of repeated recombination at domain boundaries between individual KIR genes, resulting in continual swapping of Ig domains within the family [53]. The Ig domains in the KIR family are highly conserved (90% identity), when compared with other members
of the highly promiscuous Ig domain superfamily. This evidence suggests that all human KIRs are descended from a single gene via duplication. While there has been domain swapping within the family, no insertions of domains from outside the family have occurred since its inception.
**Initial screen:** Symbol list derived from [50] and HGNC.
**References:** [50, 53­-55]

**Laminin:** Laminin is a key component of the extracellular matrix with a broad range of functions related to tissue morphogenesis. There are three Laminin subfamilies α, β, and γ. In vertebrates, peptides from these subfamilies unite in different combinations to form trimeric subcomplexes with varying functional specificity [56, 57]. Laminins are primarily composed of Laminin N, EGF Lam, Laminin B, and LamG domains repeated in tandem formation. Domain composition and the number of domain copies differs in each subfamily. The similarity in domain content is suggestive of common ancestry, followed by changes in copy number through unequal crossing over. Laminins contain a number of highly promiscuous and/or low complexity domains, including Laminin-II, EGF Lam, Laminin B, LamG, SF assemblin, Laminin N and Myosin-tail-1, that result in a large number of unrelated matches.
**Initial screen:** Laminin proteins manually identified based on SwissProt description field.
**References:** [56, 58]

**Myosin:** Myosins are a family of actin-associated molecular motors implicated in a broad range of functions, including cell polarity, cytokinesis, organelle transport, motility, muscle contraction, and, in some cases, signal transduction [59-­61]. All Myosins exhibit the same general organization: an N-terminal motor domain (the
Myosin head), a light-chain domain, and a C-terminal tail that confers specific functional properties [62­-64]. The domain content of the tail region is highly variable and can include a number of promiscuous domains (e.g., SH3, PDZ, B41) and coiled-coil regions (e.g., Myosin-tail-1). In mammals, there are at least 11 Myosin sub-classes. Each class is distinguished by a characteristic motor domain and a specific combination of C-terminal domains, implying a mode of evolution through early duplication and domain insertions, followed by additional duplications within the subclasses.
**Initial screen:** Symbol list derived from HGNC.
**References:** [62-­65]

**Notch:** Notch is a small developmental gene family. Notch genes encode multi-functional transmembrane proteins that take on a broad range of signaling functions that determine cell fate [66-­68]. Notch domain architecture consists of a series of tandem calcium-binding EGF domains in the extracellular region, several NOD domains, and a number of intracellular ankyrin repeats. Although EGF-CA and Ank are promiscuous domains, the sequence of these domains is highly conserved within the family. This family has only been observed in metazoans. The degree of conservation at both the sequence and domain architecture level indicates common ancestry.
**Initial screen:** Notch proteins are uniquely identified by gene symbols with the prefix NOTCH (HGNC).
**References:** [66­-68]

**Phosphodiesterases (PDE):** PDEs are a family of enzymes that regulate intracellular concentrations of cAMP and cGMP [69]. Since these small molecules act as intracellular mediators, PDEs indirectly influence many biological processes. Mammalian PDEs share a highly conserved C-terminal catalytic core of ~270 residues. The N-terminal regions of these proteins contain sequence motifs that determine the magnitude, duration, and location of their activity through regulation by small molecules specific to each PDE subfamily. The N-terminal portions of these proteins are highly variable due to their modular nature. However, since these regions do not contain promiscuous domains, PDEs match relatively few unrelated sequences. The PDE subfamilies arose from the same ancestral single-domain gene through repeated gene duplication and domain shuffling early in metazoan evolution [70].
**Initial screen:** Symbol list derived from HGNC.
**References:** [69­-71]

**Semaphorin (SEMA):** SEMA is a family of functionally diverse multidomain ligands. First studied in the context of neuronal cell migration during development, Semaphorins also play roles in a number of other processes related to tissue formation and restructuring during development [72, 73]. Recent evidence suggests an immunological role for Semaphorins as well. Semaphorins encode both secreted and transmembrane proteins and are characterized by a conserved extracellular core roughly 500 residues long. Five classes of Semaphorins have been identified in vertebrates. These are distinguished by their C-terminal domain structure, which confers the broad functional variation associated with this family. C-terminal domains found in Semaphorins include the promiscuous Ig and TSP1 domains. Semaphorins have been observed in metazoans and viruses, but not in yeast, protozoa or plants.
**Initial screen:** Symbol list derived from [74] and HGNC.
**References:** [74­-77]

**T-box (Tbx):** T-box transcription factors are a single-domain family of developmental genes, characterized by a conserved segment of 180-190 amino acids (the t-box) as well as C-terminal sequences required for transcriptional regulation activity [78, 79]. Both phylogenetic evidence and conserved synteny support the hypothesis that members of the Tbx family are descended from a single ancestral gene through several rounds of gene duplication [78, 80]. Tbx genes have been observed in worm, fly, mouse and human, suggesting an early metazoan origin.
**Initial screen:** Symbol list derived from [79, 81] and HGNC. If a sequence had both a symbol in our candidate list and the Tbox-specific InterPro code, IPR001699, it was placed on the preliminary roster.
**References:** [78­-81]

**Tumor Necrosis Factors (TNF):** The Tumor Necrosis Factors, acting in concert with the TNF receptors, are primarily associated with signaling pathways in the adaptive immune system [82]. They control cell proliferation and differentiation, as well as apoptosis. TNF is a single-domain family characterized by the ~150 amino acid THD
domain. Although TNFs have a single-domain, that domain is not highly conserved within the family, having only 20-30% sequence identity. Tumor Necrosis Factors evolved through repeated duplication that coincided with the emergence of the adaptive immune system and MHC expansion [83­-85]. Detailed studies, including
comparison of phylogenies, and structural and functional relationships, suggest that all vertebrate TNF proteins share a common origin with the Drosophila TNF protein [85].
**Initial screen:** Symbol list derived from [84, 86, 87] and HGNC. If a sequence had both a symbol on our candidate list and the TNF-specific InterPro code, IPR006052, it was placed on the preliminary TNF roster.
**References for TNF, TNFR and TRAF:** [82, 84, 85, 87­-92]

**Tumor Necrosis Factor Receptors (TNFR):** TNF receptors are transmembrane glycoproteins characterized by a cysteine-rich extracellular domain. Two of the three TNFR subfamilies are defined by cytoplasmic DEATH domains and TRAF interaction motifs, respectively. The third subfamily has no cytoplasmic signaling motifs. TNF receptors have low sequence conservation within the family. Phylogenetic evidence, as well as chromosomal location, supports a history of co-evolution with the TNF ligands [83­-85]. This evidence is consistent with
evolution of both families by vertical descent.
**Initial screen:** Symbol list derived from [84, 86, 87] and HGNC.
**References:** see TNF.

**Tumor necrosis factor Receptor Associated Factors (TRAF):** TRAFs mediate a broad range of functions associated with the innate and adaptive immune responses, stress response, and embryogenesis [93]. They act through many signaling molecules including, but not restricted to, TNF receptors. Most TRAFs contain a C-terminal
TRAF-specific RING finger domain, one or more zinc-finger domains, and an N-terminal MATH domain. The latter is also found in an otherwise unrelated family of metalloendopeptidases, the meprins. TRAF is an adaptable family of genes that has been recruited to varied functions over evolutionary history in response to changes
in genomic environment. Small family size, highly conserved sequence motifs, and little variation in domain architecture support descent from a single common ancestor. Although TRAF proteins mediate TNFR function, as a family they are much older [94].
**Initial screen:** TRAF proteins are uniquely identified by gene symbols with the prefix TRAF [93]
**References:** see TNF.

**Ubiquitin Specific Proteases (USP):** USPs are deubiquitinizing enzymes involved in coordinating many eukaryotic cellular processes that are mediated by modification of proteins by ubiquitin [95, 96]. These processes include cell cycle progression, DNA repair, transcriptional activation, vesicle trafficking, and signal transduction. USPs are characterized by a catalytic domain containing two highly conserved motifs responsible for catalytic activity. Outside of these conserved motifs, USP sequences vary greatly due to the broad range of substrate specificities exhibited by this family as well as diverse cellular localization signals [97]. USPs can match non-homologous
sequences with which they share a limited region of sequence similarity that confers shared functional properties (e.g., substrate specificity).
**Initial screen:** Symbol list derived from HGNC and MEROPS [98].
**References:** [95-97, 99, 100]

**Wingless-related MMTV integration site (WNT):** A highly conserved family of single-domain signaling proteins that regulate cell-cell interactions during development of the embryo [101]. There is also evidence that WNT proteins play a role in tissue homeostasis in adults. WNT homologs have been observed in fly, worm, and mammals but are absent from plants, yeast, and bacteria. The WNT family is defined in terms of conserved sequence motifs, rather than functional properties. These motifs include a specific pattern of cysteines as well as other conserved residues [101]. The observation that WNT is a conserved single-domain protein and that the WNT domain exists only in this family, supports the view that all WNT proteins arose from the same ancestor.
**Initial screen:** Symbol list derived from HGNC.
**References:** [102-­104]

**References**
1. Finn RD, Mistry J, Schuster-Bockler B, Griffiths-Jones S, Hollich V, et al. (2006) Pfam: clans, web tools and services. Nucleic Acids Res (Database issue) 34:D247­D251.
2. Mulder N, Apweiler R, Attwood T, Bairoch A, Bateman A, et al. (2005) InterPro, progress and status in 2005. Nucleic Acids Res 33:D201­-205.
3. Bairoch A, Apweiler R, Wu C, Barker W, Boeckmann B, et al. (2005) The universal protein resource (UniProt). Nucleic Acids Res 33:D154­-159.
4. Mashek D, Bornfeldt K, Coleman R, Berger J, Bernlohr D, et al. (2004) Revised nomenclature for the mammalian long-chain acyl-CoA synthetase gene family. J Lipid Res 45:1958-19­61.
5. Shockey J, Fulda M, Browse J (2002) *Arabidopsis* contains nine long-chain acyl-coenzyme A synthetase genes that participate in fatty acid and glycerolipid metabolism. Plant Physiol 129:1710­1722.
6. Coleman R, Lewin T, Van Horn C, Gonzalez-Baro M (2002) Do long-chain acyl-CoA synthetases regulate fatty acid entry into synthetic versus degradative pathways? J Nutr 132:2123­-2126.
7. Seals D, Courtneidge S (2003) The ADAMs family of metalloproteases: multidomain proteins with multiple functions. Genes Dev 17:7-­30.
8. Stone A, Kroeger M, Sang Q (1999) Structure-function analysis of the ADAM family of disintegrin-like and metalloproteinase-containing proteins (review). J Protein Chem 18:447­-465.
9. Wolfsberg T, White J (1996) ADAMs in fertilization and development. Dev Biol 180:389­-401.
10. Wolfsberg T, Primakoff P, Myles D, White J (1995) Adam, a novel family of membrane proteins containing a disintegrin and metalloprotease domain: multipotential functions in cell-cell and cell-matrix interactions. J Cell
Biol 131:275­-278.
11. Wolfsberg T, Straight P, Gerena R, Huovila A, Primakoff P, et al. (1995) ADAM, a widely distributed and developmentally regulated gene family encoding membrane proteins with a disintegrin and metalloprotease domain. Dev Biol 169:378­-383.
12. Bates E, Fridman W, Mueller C (2002) The ADAMDEC1 (decysin) gene structure: evolution by duplication in a metalloprotease gene cluster on chromosome 8p12. Immunogenetics 54:96-­105.
13. Wharton K (2003) Runnin’ with the Dvl: proteins that associate with Dsh/Dvl and their significance to Wnt signal transduction. Dev Biol 253:1­-17.
14. Sheldahl L, Slusarski D, Pandur P, Miller J, Khl M, et al. (2003) Dishevelled activates Ca2+ flux, PKC, and CamKII in vertebrate embryos. J Cell Biol 161:769­-777.
15. Ornitz D, Itoh N (2001) Fibroblast growth factors. Genome Biol 2:REVIEWS3005.
16. Itoh N, Ornitz D (2004) Evolution of the Fgf and Fgfr gene families. Trends Genet 20:563­-569.
17. Coulier F, Pontarotti P, Roubin R, Hartung H, Goldfarb M, et al. (1997) Of worms and men: An evolutionary perspective on the fibroblast growth factor (FGF) and FGF receptor families. J Mol Evol 44:43­-56.
18. Granadino B, Perez-Sachez C, Rey-Campos J (2000) Fork head transcription factors. Current Genomics 1:1710­-1722.
19. Kaestner K, Knochel W, Martinez D (2000) Unified nomenclature for the winged helix/forkhead transcription factors. Genes Dev 14:142­-146.
20. Adell T, Muller WEG (2004) Isolation and characterization of five Fox (Forkhead) genes from the sponge *Suberites domuncula*. Gene 334:35-­46.
21. Mazet F, Yu J, Liberles D, Holland L, Shimeld S (2003) Phylogenetic relationships of the fox (Forkhead) gene family in the bilateria. Gene 316:79­-89.
22. Patient R, McGhee J (2002) The GATA family (vertebrates and invertebrates). Curr Opin Genet Dev 12:416-4­22.
23. Lowry J, Atchley W (2000) Molecular evolution of the GATA family of transcription factors: conservation within the DNA-binding domain. J Mol Evol 50:103-­115.
24. Hanks S (2003) Genomic analysis of the eukaryotic protein kinase superfamily: a perspective. Genome Biol 4:111.
25. Shiu S, Li W (2004) Origins, lineage-specific expansions, and multiple losses of tyrosine kinases in eukaryotes. Mol Biol Evol 21:828­-840.
26. Cheek S, Zhang H, Grishin N (2002) Sequence and structure classification of kinases. J Mol Biol 320:855­-881.
27. Scheeff ED, Bourne PE (2005) Structural evolution of the protein kinase-like superfamily. PLoS Comput Biol 1:e49.
28. Marchler-Bauer A, Anderson J, Cherukuri P, DeWeese-Scott C, Geer L, et al. (2005) CDD: a Conserved Domain Database for protein classification. Nucleic Acids Res 33:D192-19­6.
29. Tordai H, Nagy A, Farkas K, Banyai L, Patthy L (2005) Modules, multidomain proteins and organismic complexity. FEBS J 272:5064­-5078.
30. Manning G, Whyte D, Martinez R, Hunter T, Sudarsanam S (2002) The protein kinase complement of the human genome. Science 298:1912-­1934.
31. Robinson D, Wu Y, Lin S (2000) The protein tyrosine kinase family of the human genome. Oncogene 19:5548­-5557.
32. Gu J, Gu X (2003) Natural history and functional divergence of protein tyrosine kinases. Gene 317:49­57.
33. Gu J, Wang Y, Gu X (2002) Evolutionary analysis for functional divergence of jak protein kinase domains and tissue-specific genes. J Mol Evol 54:725-­733.
34. Manning G, Plowman G, Hunter T, Sudarsanam S (2002) Evolution of protein kinase signaling from yeast to man. Trends Biochem Sci 27:514­-520.
35. Krupa A, Srinivasan N (2002) The repertoire of protein kinases encoded in the draft version of the human genome: atypical variations and uncommon domain combinations. Genome Biol 3:RESEARCH0066.
36. Kostich M, English J, Madison V, Gheyas F, Wang L, et al. (2002) Human members of the eukaryotic protein kinase family. Genome Biol 3:0043.1­0043.12.
37. Nars M, Vihinen M (2001) Coevolution of the domains of cytoplasmic tyrosine kinases. Mol Biol Evol 18:312­-321.
38. Hanks S, Hunter T (1995) The eukaryotic protein kinase superfamily: kinase (catalytic) domain structure and classification. FASEB J 9:576­-596.
39. Rousset D, Agnes F, Lachaume P, Andre C, Galibert F (1995) Molecular evolution of the genes encoding receptor tyrosine kinase with immunoglobulinlike domains. J Mol Evol 41:421­-429.
40. Hanks S, Quinn A (1991) Protein kinase catalytic domain sequence database: identification of conserved features of primary structure and classification of family members. Methods Enzymol 200:38­-62.
41. Hanks S, Quinn A, Hunter T (1988) The protein kinase family: conserved features and deduced phylogeny of the catalytic domains. Science 241:42­-52.
42. Miki H, Setou M, Hirokawa N (2003) Kinesin superfamily proteins (KIFs) in the mouse transcriptome. Genome Res 13:1455­-1465.
43. Miki H, Okada Y, Hirokawa N (2005) Analysis of the kinesin superfamily: insights into structure and function. Trends Cell Biol 15:467-­476.
44. Reddy A, Day I (2001) Kinesins in the Arabidopsis genome: a comparative analysis among eukaryotes. BMC Genomics 2:2­-14.
45. Iwabe N, Miyata T (2002) Kinesin-related genes from diplomonad, sponge, amphioxus, and cyclostomes: divergence pattern of kinesin family and evolution of giardial membrane-bounded organella. Mol Biol Evol 19:1524­-1533.
46. Lawrence C, et al. (2004) A standardized kinesin nomenclature. J Cell Biol 67:19-­22.
47. Dagenbach E, Endow S (2004) A new kinesin tree. J Cell Sci 117:3-­7.
48. Miki H, Setou M, Kaneshiro K, Hirokawa N (2001) All kinesin superfamily protein, KIF, genes in mouse and human. Proc Natl Acad Sci U S A 98:7004­-7011.
49. Kim A, Endow S (2000) A kinesin family tree. J Cell Sci 113 Pt 21:3681-368­2.
50. Yawata M, Yawata N, Abi-Rached L, Parham P (2002) Variation within the human killer cell immunoglobulin-like receptor (KIR) gene family. Crit Rev Immunol 22:463­-482.
51. Welch A, Kasahara M, Spain L (2003) Identification of the mouse killer immunoglobulin-like receptor-like (Kirl) gene family mapping to chromosome X. Immunogenetics 54:782-­790.
52. Anderson S, Ortaldo J, McVicar D (2001) The ever-expanding Ly49 gene family: repertoire and signaling. Immunol Rev 181:79­-89.
53. Rajalingam R, Parham P, Abi-Rached L (2004) Domain shuffling has been the main mechanism forming new hominoid killer cell Ig-like receptors. The Journal of Immunology 172:356­-369.
54. Sambrook J, Bashirova A, Palmer S, Sims S, Trowsdale J, et al. (2005) Single haplotype analysis demonstrates rapid evolution of the killer immunoglobulin-like receptor (KIR) loci in primates. Genome Research 15:25-­35.
55. Radaev S, Sun P (2003) Structure and function of natural killer cell surface receptors. Annu Rev Biophys Biomol Struct 32:93­-114.
56. Engel J (1992) Laminins and other strange proteins. Biochemistry 31:10643­-10651.
57. Hutter H, Vogel B, Plenefisch J, Norris C, Proenca R, et al. (2000) Conservation and novelty in the evolution of cell adhesion and extracellular matrix genes. Science 287:989­-994.
58. Miner J, Yurchenco P (2004) Laminin functions in tissue morphogenesis. Annu Rev Cell Dev Biol 20:255­-284.
59. Richards T, Cavalier-Smith T (2005) Myosin domain evolution and the primary divergence of eukaryotes. Nature 436:1113­-1118.
60. Goodson H, Dawson S (2006) Multiplying myosins. Proc Natl Acad Sci U S A 103:3498­-3499.
61. Foth B, Goedecke M, Soldati D (2006) New insights into myosin evolution and classification. Proc Natl Acad Sci U S A 103:3681­-3686.
62. Berg J, Powell B, Cheney R (2001) A millennial myosin census. Mol Biol Cell 12:780-7­94.
63. Sellers J (2000) Myosins: a diverse superfamily. Biochem Biophys Acta 1496:3­-22.
64. Thompson R, Langford G (2002) Myosin superfamily evolutionary history. Anat Rec 268:276­-289.
65. Korn E (2000) Coevolution of head, neck, and tail domains of myosin heavy chains. Proc Natl Acad Sci U S A 97:12559­-12564.
66. Maine E, Lissemore J, Starmer W (1995) A phylogenetic analysis of vertebrate and invertebrate Notch-related genes. Mol Phylogenet Evol 4:139­-149.
67. Westin J, Lardelli M (1997) Three novel Notch genes in zebrafish: implications for vertebrate Notch gene evolution and function. Dev Genes Evol 207:51-­63.
68. Kortschak R, Tamme R, Lardelli M (2001) Evolutionary analysis of vertebrate Notch genes. Dev Genes Evol 211:350­-354.

69. Degerman E, Belfrage P, Manganiello V (1997) Structure, localization, and regulation of cGMP-inhibited phosphodiesterase (PDE3). J Biol Chem 272:6823­-6826.
70. Koyanagi M, Suga H, Hoshiyama D, Ono K, Iwabe N, et al. (1998) Ancient gene duplication and domain shuffling in the animal cyclic nucleotide phosphodiesterase family. FEBS Lett 436:323-32­8.
71. Beavo J, Brunton L (2002) Cyclic nucleotide research--still expanding after half a century. Nat Rev Mol Cell Biol 3:710­-718.
72. Raper J (2000) Semaphorins and their receptors in vertebrates and invertebrates. Curr Opin Neurobiol 10:88­94.
73. Yazdani U, Terman J (2006) The semaphorins. Genome Biol 7:211.
74. Goodman C, Kolodkin A, Luo Y, Püschel A, Raper J (1999) Unified nomenclature for the semaphorins/collapsins. Cell 97:551-­552.
75. Pasterkamp R, Kolodkin A (2003) Semaphorin junction: making tracks toward neural connectivity. Curr Opin Neurobiol 13:79-­89.
76. Kumanogoh A, Kikutani H (2003) Immune semaphorins: a new area of semaphorin research. J Cell Sci 116:3463-34­70.
77. Kikutani H, Kumanogoh A (2003) Semaphorins in interactions between T cells and antigen-presenting cells. Nat Rev Immunol 3:159­-167.
78. Agulnik S, Garvey N, Hancock S, Ruvinsky I, Chapman D, et al. (1996) Evolution of mouse T-box genes by tandem duplication and cluster dispersion. Genetics 144:249­-254.
79. Wilson V, Conlon F (2002) The T-box family. Genome Biol 3:REVIEWS3008.
80. Ruvinsky I, Silver L, Gibson-Brown J (2000) Phylogenetic analysis of T-box genes demonstrates the importance of amphioxus for understanding evolution of the vertebrate genome. Genetics 156:1249-­1257.
81. Papaioannou V, Silver L (1998) The T-box gene family. Bioessays 20:9­-19.
82. Bodmer J, Schneider P, Tschopp J (2002) The molecular architecture of the TNF superfamily. Trends Biochem Sci 27:19­-26.
83. Locksley R, Killeen N, Lenardo M (2001) The TNF and TNF receptor superfamilies: integrating mammalian biology. Cell 104:487­-501.
84. MacEwan D (2002) TNF ligands and receptors--a matter of life and death. Br J Pharmacol 135:855-­875.
85. Collette Y, Gilles A, Pontarotti P, Olive D (2003) A co-evolution perspective of the TNFSF and TNFRSF families in the immune system. Trends Immunol 24:387­-394.
86. Ware C (2003) The TNF superfamily. Cytokine Growth Factor Rev 14:181­-184.
87. Locksley R, Killeen N, Lenardo M (2001) The TNF and TNF receptor superfamilies: integrating mammalian biology. Cell 104:487­-501.
88. Dempsey P, Doyle S, He J, Cheng G (2003) The signaling adaptors and pathways activated by TNF superfamily. Cytokine Growth Factor Rev 14:193-­209.
89. Abi-Rached L, Gilles A, Shiina T, Pontarotti P, Inoko H (2002) Evidence of en bloc duplication in vertebrate genomes. Nat Genet 31:100­-105.

90. Granger S, Butrovich K, Houshmand P, Edwards W, Ware C (2001) Genomic characterization of LIGHT reveals linkage to an immune response locus on chromosome 19p13.3 and distinct isoforms generated by alternate
splicing or proteolysis. J Immunol 167:5122-512­8.
91. Bays M, Hartung A, Ezer S, Pispa J, Thesleff I, et al. (1998) The anhidrotic ectodermal dysplasia gene (EDA) undergoes alternative splicing and encodes ectodysplasin-A with deletion mutations in collagenous repeats. Hum
Mol Genet 7:1661­-1669.
92. Srivastava A, Pispa J, Hartung A, Du Y, Ezer S, et al. (1997) The Tabby phenotype is caused by mutation in a mouse homologue of the EDA gene that reveals novel mouse and human exons and encodes a protein (ectodysplasin-A) with collagenous domains. Proc Natl Acad Sci U S A 94:13069­-13074.
93. Inoue J, Ishida T, Tsukamoto N, Kobayashi N, Naito A, et al. (2000) Tumor necrosis factor receptor-associated factor (TRAF) family: adapter proteins that mediate cytokine signaling. Exp Cell Res 254:14-­24.
94. Grech A, Quinn R, Srinivasan D, Badoux X, Brink R (2000) Complete structural characterisation of the mammalian and *Drosophila* TRAF genes: implications for TRAF evolution and the role of RING finger splice variants. Mol Immunol 37:721­-734.
95. Wing S (2003) Deubiquitinating enzymes--the importance of driving in reverse along the ubiquitin-proteasome pathway. Int J Biochem Cell Biol 35:590­-605.
96. Kim J, Park K, Chung S, Bang O, Chung C (2003) Deubiquitinating enzymes as cellular regulators. J Biochem (Tokyo) 134:9­-18.
97. Quesada V, Diaz-Perales A, Gutierrez-Fernandez A, Garabaya C, Cal S, et al. (2004) Cloning and enzymatic analysis of 22 novel human ubiquitin-specific proteases. Biochem Biophys Res Commun 314:54-­62.
98. Rawlings N, Tolle D, Barrett A (2004) MEROPS: the peptidase database. Nucleic Acids Res 32:D160­-164.
99. Burrows J, McGrattan M, Johnston J (2005) The DUB/USP17 deubiquitinating enzymes, a multigene family within a tandemly repeated sequence. Genomics 85:524­-529.
100. Holowaty M, Sheng Y, Nguyen T, Arrowsmith C, Frappier L (2003) Protein interaction domains of the ubiquitin-specific protease, USP7/HAUSP. J Biol Chem 278:47753­-47761.
101. Nusse R (2005) Wnt signaling in disease and in development. Cell Research 15:28-­32.
102. Schubert M, Holland L, Holland N, Jacobs D (2000) A phylogenetic tree of the Wnt genes based on all available full-length sequences, including five from the cephalochordate amphioxus. Mol Biol Evol 17:1896-­903.
103. Cadigan K (2002) Wnt signaling-20 years and counting. Trends Genet 18:340­-342.
104. Logan C, Nusse R (2004) The Wnt signaling pathway in development and disease. Annu Rev Cell Dev Biol 20:781-­810.

1. HGNC: http//www.gene.ucl.ac.uk/nonmenclature/genefamily.html [↑](#footnote-ref-2)
